# High-Pressure Phase Behavior of α-Olefin + *n*-Hexane + Ethylene/1-Octene Copolymer Systems: Experimental Study and Modeling

**DOI:** 10.3390/polym18010064

**Published:** 2025-12-25

**Authors:** Ruijun Zhang, Ziyi Dong, Qiqi He, Junhua Li, Yuexin Hu, Jianhua Qian

**Affiliations:** 1 College of Chemistry and Chemical Engineering, China University of Petroleum (East China), Qingdao 266555, China; zhangruijun523@gmail.com (R.Z.); qqhe65878@gmail.com (Q.H.); 2 College of Petrochemical Engineering, Liaoning Petrochemical University, Fushun 113001, China; 13898210007@163.com (Z.D.); lijunhua0521@163.com (J.L.); yxhu1981@163.com (Y.H.)

**Keywords:** phase behavior, α-olefin, hexane, ethylene/1-octene copolymer, modified Sanchez-Lacombe equation of state

## Abstract

Accurate knowledge of phase behavior in polyolefin–solvent mixtures is critical for ensuring stable operation and safe scale-up of industrial solution polymerization processes. The binary (*n*-hexane + ethylene/1-octene copolymer, POE96k-10) and ternary (α-olefin + *n*-hexane + POE96k-10) phase behaviors were investigated via a visual high-pressure cell (POE96k-10: *M*_w_ = 96 kg·mol^–1^, *M*_w_/*M*_n_ = 3.87, 1-octene mole fraction = 10.31 mol%) at temperatures of 380~480 K and pressures as high as 14 MPa. To systematically analyze the effects of α-olefin mass fraction and type on phase transition, four industrially relevant α-olefins (ethylene, 1-butene, 1-hexene, and 1-octene) were investigated. The results show that the phase transition temperature and pressure for liquid–liquid and liquid–vapor transitions show an approximately linear dependence on α-olefin mass fraction. Ethylene, 1-butene, and 1-hexene lower the phase transition temperature, whereas 1-octene increases it. Ethylene exhibits a strong anti-solvent effect, significantly lowering the transition temperature while increasing the phase transition pressure. The modified Sanchez-Lacombe equation of state (MSL EOS) effectively correlates and reproduces the phase equilibrium data of the α-olefin + *n*-hexane + POE96k-10 ternary systems, though its accuracy decreases with increasing α-olefin chain length.

## 1. Introduction

Polyolefin elastomers (POEs) are copolymers derived from ethylene and α-olefins, which typically contain more than 20 wt% α-olefins [1,2,3]. Combining the advantages of plastics and rubber, POEs are widely used in automotive components, impact-resistant modifications, wires and cables, and photovoltaic encapsulation films [4,5]. POEs are synthesized via high-temperature solution polymerization, in which ethylene and comonomers polymerize in an alkane solvent with a metallocene catalyst. After polymerization, the polymer solution undergoes sequential medium- and low-pressure separations to remove the solvent and light components, followed by melt pelletization. Maintaining a single liquid phase before entering the flash drum is critical. Liquid–liquid phase separation can reduce polymer solubility, disrupt process stability, and cause polymer deposition inside the equipment [6,7]. This behavior increases cleaning frequency and unplanned downtime. Therefore, understanding the phase behavior of POE solution under high pressure is essential for optimizing separation, ensuring production stability, and developing predictive model [8,9].

**Scheme 1:**
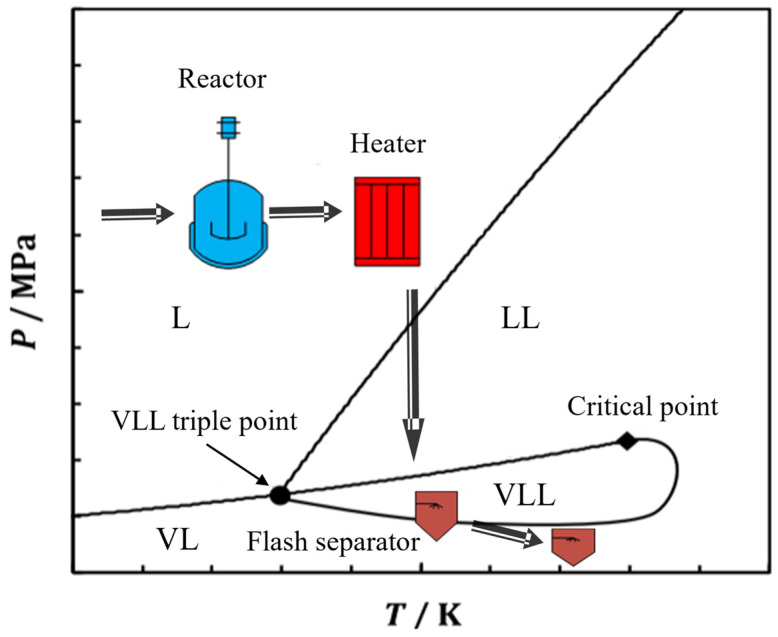
Schematic diagram of solution polymerization process.

The solvent + polyolefin system used in solution processes typically exhibits low critical solution temperature (LCST) behavior (Scheme 1) [10,11]. This phase diagram consists of a gas phase, distinct liquid phases with varying compositions, and a homogeneous single liquid phase. The liquid–liquid (LL) line divides the homogeneous liquid phase and the LL zone, while the vapor-liquid (VL) line marks the transition to the VL area.

The phase behavior of solvent + polyolefin systems plays a crucial role in solution-based polyolefin production. Extensive experimental research has been carried out to investigate the phase transition of polyolefin solutions [12,13,14]. Chen et al. [15] examined the phase equilibrium of *n*-hexane + polyethylene (PE) and ethylene + *n*-hexane + PE mixtures at 370–480 K and pressures reaching 20 MPa, revealing how polymer molecular weight affects phase separation. Nagy et al. [16] investigated the LL and VL curves for *n*-hexane + LLDPE binary and ethylene + *n*-hexane + LLDPE ternary mixtures at 400–500 K. Haruki et al. [8] studied phase separation in hexane + PE systems, demonstrating that higher PE polydispersity led to lower polymer solubility at the maximum separation pressure. Sun [9] conducted extensive high-pressure measurements on 32 polyolefins in three different alkane solvents, mapping their liquid–liquid equilibrium (LLE) boundaries. Tork [17] reported phase behavior data for an ethylene + 1-octene + *n*-hexane + HDPE system, showing that each wt% of 1-octene in the solvent reduced the phase transition pressure by approximately 0.07 MPa. Haruki et al. [18,19] also studied the phase characteristics of hydrogenated polybutadienes and LLDPE in ethylene + 1-hexene + *n*-hexane systems, highlighting the influence of ethylene, 1-hexene, and polymer content. They found that 1-hexene had minimal impact due to its chemical similarity to *n*-hexane. Additional studies by Swanepoel et al. [20] further explored how α-olefin content influences the phase dynamics of PE-based systems.

To model phase behavior, thermodynamic equations are commonly employed to correlate experimental data and reproduce phase transitions beyond the data. The modified Sanchez-Lacombe (MSL) equation of state (EOS) was developed by Krenz et al. [21] as an extension of the original Sanchez-Lacombe (SL) equation [22,23,24,25]. It incorporates Neau’s residual Helmholtz energy to ensure correct behavior in the low-pressure limit [26]. Gauter and Heidemann [27] subsequently applied the volume translation factor proposed by Péneloux et al. [28] to the MSL equation of state and proposed a parameterization scheme that enables the calculation of the MSL parameters from the critical properties of individual components. In comparison with the PC-SAFT equation of state [29,30,31], the MSL approach offers improved flexibility and computational efficiency for modeling (solvent + polyolefin) systems. Therefore, this study employs the MSL EOS to model the phase equilibrium of POE solutions.

Although extensive studies have been conducted on polyethylene and LLDPE solutions, systematic phase-behavior data for POE systems, particularly in the presence of different α-olefins, remain relatively limited. Given that comonomers constitute a significant fraction of the solvent phase in industrial solution polymerization processes, it is therefore of practical interest to extend existing phase-behavior studies to POE systems and to quantify the trends induced by different α-olefins. Building on the phase transition data of the *n*-hexane + POE96k-10 binary system, this study further examines the phase transitions in the α-olefin + *n*-hexane + POE96k-10 ternary system. The objective is to systematically investigate how α-olefin type and mass fraction influence liquid–liquid and liquid–vapor phase transitions in polyolefin elastomer solutions, thereby providing trend-based insights that are relevant for process analysis and operation.

## 2. Experiment

### 2.1. Materials

High-purity ethylene and 1-butene were purchased from Shenyang Shuntai Special Gas Co., Ltd., Shenyang, China (Shuntai Gas) and used as received without further purification. *n*-Hexane, 1-hexene, and 1-octene were obtained from Shanghai Aladdin Scientific Corporation, Shanghai, China (Aladdin Scientific, Shanghai, China) and dried over molecular sieves prior to use. High-purity nitrogen gas from Shuntai Gas was used for purging and displacement of the phase equilibrium cell, while industrial-grade xylene from Aladdin Scientific was employed for cleaning the cell. Detailed information on all chemicals used is provided in Table 1.

The POE sample was obtained from Dow Chemical. This polymer was synthesized via metallocene catalysis using ethylene and 1-octene as monomers. The molecular weight and distribution (MWD) of the POE sample were analyzed with high-temperature Gel Permeation Chromatography (HT-GPC, Polymer Char, Valencia City, Spain), equipped with infrared and differential viscosity detectors. 1,2,4-Trichlorobenzene was used as the solvent with a flow rate of 1.0 mL·min^−1^, and the dissolution time was set to 90 min to ensure complete dissolution of the polymer. Universal calibration was performed using narrow molecular weight distribution polystyrene standards. The 1-octene incorporation rate was measured by ^13^C-NMR, performed on a JEOL JNM-ECZL400S NMR spectrometer (JEOL, Japan) at 125 °C, according to the method outlined in the literature [32,33]. The polymer was dissolved in a 10 wt% deuterated ortho-dichlorobenzene (o-DCB) solution at 150 °C and maintained for 3 h to ensure complete dissolution and homogeneity. The NMR experiments were conducted using a 90° pulse angle with inverse-gated proton decoupling (decoupled spectrum without NOE), an acquisition time of 1.3 s, a pulse delay of 8 s (Rd > 5T_1_), and a spectral width of 8000 Hz. Each spectrum was acquired by averaging at least 5000 scans. The characterization outcomes of the POE sample are listed in Table 2. The sample is designated as POE96k-10, where “96k” signifies the weight-average molecular weight of roughly 96 kg·mol^–1^, and “10” indicates the molar percentage of 1-octene content.

### 2.2. Experimental Device

**Figure 1:**
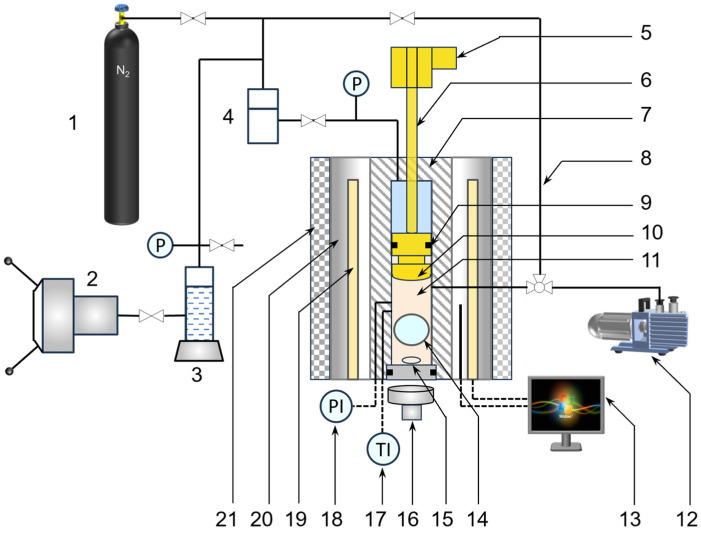
Illustration of the high-pressure phase equilibrium measurement apparatus. (1) Nitrogen cylinder; (2) hand pump; (3) buffer container; (4) back pressure valve; (5) gear motor; (6) lead screw with scale; (7) cell body; (8) nitrogen line; (9) sealing O-rings; (10) movable piston; (11) high-pressure cell; (12) vacuum pump; (13) temperature controller; (14) sapphire window; (15) magnetic stirrer bar; (16) magnetic stirrer; (17) temperature indicator; (18) pressure indicator; (19) tubular heater; (20) aluminum block; (21) insulation layer.

The setup depicted in Figure 1 is classified as a high-pressure phase behavior device using the synthetic method, as reported in the literature [34,35]. In the synthetic method, a mixture with a defined composition is created and placed into a cell. Phase separation is studied under varying conditions and the emergence of new phases is detected through visual inspection or techniques such as light scattering.

The equipment includes a variable-volume high-pressure cell equipped with a viewing window and a stirring mechanism, along with a backpressure regulator system. The cell has a 50 mL volume, operates between 0 and 20 MPa pressure, and 298–500 K temperature. A magnetic stirrer ensures the solution remains homogeneous in the cell. The cell is enclosed within a heated cylindrical aluminum block, which is insulated with glass wool to minimize heat loss. For accurate temperature measurement, a platinum resistance thermometer (FLUKE, Model 5622-10) measures the temperatures of both the aluminum block and the cell, with a measurement uncertainty of ±0.04 K.

The apparatus provides two pressure regulation methods. The first method employs a geared motor to drive a lead screw, which adjusts the piston’s position and thereby controls the cell volume. The second method uses a manual pump to inject liquid into the space above the piston’s head, regulating the system pressure. The pressure inside the cell is monitored by a sensor in direct contact with the solution, with readings displayed on a pressure indicator (ONEHalf20, Model IP148). The manufacturer calibrates the pressure gauge using a deadweight tester, which has a calibration uncertainty of ±0.02 MPa. Phase behavior in the cell is observed through a sapphire window with a borescope connected to a high-definition camera. A halogen lamp positioned outside the opposite sapphire window illuminates the cell interior, enhancing visibility of phase transitions.

### 2.3. Experimental Procedure

#### 2.3.1. System Loading

The mass of POE96k-10 particles was weighed on an electronic balance (METTLER XPR226DRQ/AC, resolution 0.01 mg) and then transferred into the cell. Liquid components (*n*-hexane and α-olefin) were degassed via freeze–thaw, mixed at the desired α-olefin fraction, and stored in a vial. To prevent degradation and polymerization, 500 mg·kg^–1^ butylated hydroxytoluene (BHT) was added. The liquid mixture mass was determined by weight difference before transfer into the cell. Gaseous components (ethylene or 1-butene) were introduced in a similar manner.

#### 2.3.2. Phase Transition Measurement

After introducing all components, the polymer was dissolved and thoroughly homogenized at 400 K and 10 MPa and subsequently equilibrated for two hours. The liquid–vapor transition (L → VL) pressure was recorded by gradually decreasing the pressure until bubbles appeared, with a variation of no more than 0.03 MPa over three trials. The LL to VLL phase transition (LL → VLL) was determined in a similar manner, starting from a heterogeneous liquid phase.

The measurement of liquid–liquid transition (L → LL) starts at least 1 MPa above the expected transition pressure, with pressure reductions in increments not exceeding 0.2 MPa. Once the pressure stabilizes, the liquid is monitored for the formation of cloud-like streaks, and the pressure at which they first appear is noted as liquid–liquid transition pressure. Subsequently, the pressure is increased by 0.1 MPa to verify that the cloud-like streaks disappear. This process is conducted a minimum of three times to confirm the recorded cloud point pressure varies by no more than 0.1 MPa. After each measurement, the solution is re-homogenized, and the temperature is adjusted for the next test.

## 3. Uncertainty Analysis

Uncertainty analysis was conducted to quantify the uncertainty associated with each experimental data point. In this study, uncertainties are expressed as combined standard uncertainties ($u_{c}$) and were evaluated following the Guide to the Expression of Uncertainty in Measurement (GUM) [36]. The present section provides only a brief overview of the uncertainties in temperature, pressure, and composition, whereas detailed uncertainty estimation procedures and complete uncertainty budgets are given in the Supporting Information.

### 3.1. Uncertainty of Temperature

The combined standard uncertainty of temperature measurement, *u*_c_(*T*) = 0.35 °C, mainly includes the following contributions: the calibration uncertainty of the temperature sensor indicator (0.19 °C), temperature measurement fluctuations (<0.2 °C), the accuracy of the temperature sensor indicator (0.5 °C), resolution (0.1 °C), and hysteresis (0.002 °C).

### 3.2. Uncertainty of Pressure

For the L → VL and LL → VLL phase transitions, the combined standard uncertainty of pressure measurement, *u*_c_(*P*_meas_), is 0.03 MPa, whereas for the L → LL phase transition, this uncertainty is 0.04 MPa. The main sources of uncertainty include (1) the uncertainty arising from the accuracy (*p* = 0.03 MPa) and resolution (*r* = 0.01 MPa) of the pressure sensor indicator; (2) hysteresis error due to the maximum hysteresis (0.03 MPa); (3) pressure calibration uncertainty (0.02 MPa); and (4) pressure measurement fluctuations (<0.1 MPa).

The uncertainty *u*_c_(*P*_meas_) accounts only for pressure measurement. The phase-transition pressure is additionally affected by temperature and composition; therefore, the combined standard uncertainty *u*_c_(*P*) includes the contributions from (∂*P*/∂*T*), (∂*P*/∂*w*), and the uncertainties in pressure, temperature, and composition. Since these sensitivities depend on the system and phase-transition type (see Supporting Information), *u*_c_(*P*) varies accordingly and is reported for each system in the data tables.

Typically, *u*_c_(*P*) is 0.05 MPa for L → VL and LL → VLL transitions, 0.13 MPa for L → LL transitions in systems containing ethylene or 1-butene, and 0.09 MPa for other L → LL transitions.

### 3.3. Uncertainty of Component

The component uncertainty, *u*_c_(*w*), was calculated from the uncertainties in the masses of the compounds charged into the phase-equilibrium cell, *u*_c_(*m*). Because different compounds were used, the resulting uncertainties vary and are reported in the corresponding experimental data tables. Although compound purity was considered in the uncertainty analysis, its contribution to the final composition uncertainty is negligible due to the high purity of the materials employed. Detailed uncertainty estimation procedures are provided in the Supporting Information.

### 3.4. Uncertainty of LCST Determination

The lower critical solution temperature (LCST) was determined using the phase-boundary interpolation method proposed by Irani and Kiran, in which the LCST is defined as the intersection of two empirically fitted phase boundaries in the pressure–temperature (*P*–*T*) space. Quadratic polynomials (*P* = *aT*^2^ + *bT* + *c*) were fitted to the L → LL transition data and to the combined L → VL and LL → VLL transition data, respectively, both exhibiting excellent goodness of fit (*R*_adj_^2^ > 0.99). A sensitivity analysis comparing quadratic and cubic polynomial representations showed that the resulting LCST temperature and pressure differed by less than the estimated uncertainty, indicating that the LCST determination is insensitive to the polynomial order (further details are provided in the Supporting Information).

The uncertainty of the LCST was quantified using a Monte Carlo–based error propagation approach, in which the fitted polynomial coefficients were randomly sampled according to their statistical uncertainties and the intersection point was repeatedly calculated (see Supporting Information for details). The mean values of the resulting LCST temperature and pressure distributions were reported as the LCST, while the corresponding expanded uncertainties were evaluated using a coverage factor of k = 2, corresponding to a 95% confidence level. Overall, the uncertainty of T_LCST_ is approximately 4.5 K, and that of P_LCST_ is about 0.40 MPa; detailed values for different systems are listed in Table S7.

## 4. Thermodynamic Model

The pressure equation of the MSL EOS is given as follows [37]:

(1) $$\frac{P}{RT}=\frac{1-d}{v}-\frac{d}{b}\ln\left(\frac{v-b}{v}\right)-\frac{a}{v^{2}RT}$$

In this context, *v* represents the molar volume adjusted using the Péneloux volume shift factor *c*, where *v* = *v*_t_ − *c*, with *v*_t_ denoting the uncorrected molar volume. For multicomponent systems, the MSL EOS incorporates four parameters, which are determined through the following mixing rules [37]:

(2) $$a=\sum_{i=1}^{N_{A}}\sum_{j=1}^{N_{A}}x_{i}x_{j}M_{i}M_{j}a_{ij}\left(1-k_{ij}\right)$$

(3) $$b=\sum_{i=1}^{N_{A}}x_{i}M_{i}b_{i}$$

(4) $$c=\sum_{i=1}^{N_{A}}x_{i}M_{i}c_{i}$$

(5) $$d=\sum_{i=1}^{N_{A}}x_{i}M_{i}d_{i}$$

Here, *a_ij_* and *b_i_* represent combinations of pure compound parameters:

(6) $$a_{ij}=\frac{d_{i}d_{j}\left(\sqrt{\varepsilon_{i}\varepsilon_{j}}\right)\left(v_{i}^{*}+v_{j}^{*}\right)}{2}$$

(7) $$b_{i}=d_{i}v_{i}^{*}$$

The MSL EOS needs four parameters for every pure compound: lattice energy, *ε_i_*; the volume per lattice site, $v_{i}^{*}$; the number of lattice sites, *d_i_*; and the volume shift parameter, *c_i_*, along with the binary interaction parameter, *k_ij_*, which accounts for interactions between any two compounds. Given the critical properties of a single substance, *ε_i_*, $v_{i}^{*}$, *d_i_*, and *c_i_* can be calculated using the parameterization approach proposed by Gauter and Heidemann [27]. In this work, the MSL parameters for POE96k-10 were derived from the LLDPE parameters reported in the literature [38]. This treatment represents an effective first-order approximation, since within the MSL framework these parameters primarily reflect segment-level average energetic and volumetric properties of the polyethylene backbone, while the influence of moderate short-chain branching in nonpolar ethylene/α-olefin copolymers is generally regarded as a second-order effect. Nevertheless, this parameter transfer does not explicitly capture local packing effects induced by branching and is therefore expected to be most reliable for POE systems with similar backbone chemistry and comparable comonomer contents. Table S1 in Supporting Information presents the critical properties of the substances used in this research, as well as the MSL equation parameters derived from them.

For polymer–solvent binary interaction parameters, *k_ij_* is assumed to exhibit a linear dependence on temperature within the investigated temperature window, as given in Equation (8). Such a linear temperature-dependent form has been widely adopted in the literature for polymer–solvent systems [7,8,18,19,37,38], as it provides an improved representation of liquid–liquid phase boundaries. Accordingly, the present linear expression is employed as an empirical first-order approximation. The methods for determining $k_{ij}^{a}$ and $k_{ij}^{b}$ are presented in Section 5.3.

(8) $$k_{ij}(T)=k_{ij}^{a}+k_{ij}^{b}T$$

**Figure 2:**
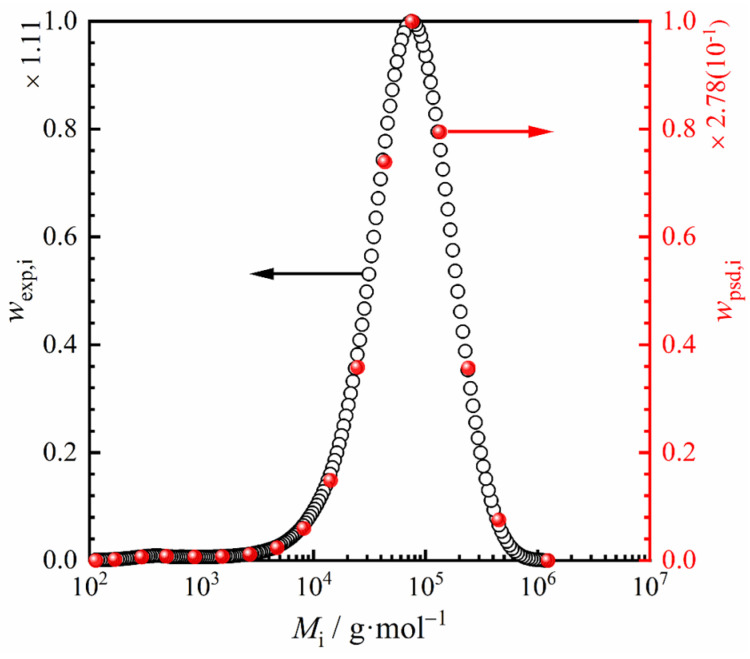
Molecular weight distribution (MWD) and discrete pseudocomponent distribution of POE96k-10. (◯) Experimental MWD from HT-GPC; (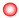) pseudocomponents from Table 3.

When applying the MSL equation, the polymer is discretized into multiple pseudocomponents to represent its molecular weight and distribution. In this work, POE96k-10 was represented using 17 pseudocomponents through the anchored integration method [21]. Detailed distribution data are presented in Figure 2 and Table 3. The phase equilibrium data modeling results in the following section demonstrate that these 17 pseudocomponents effectively represent POE96k-10.

## 5. Results and Discussion

### 5.1. Hexane + POE96k-10 Systems

**Figure 3:**
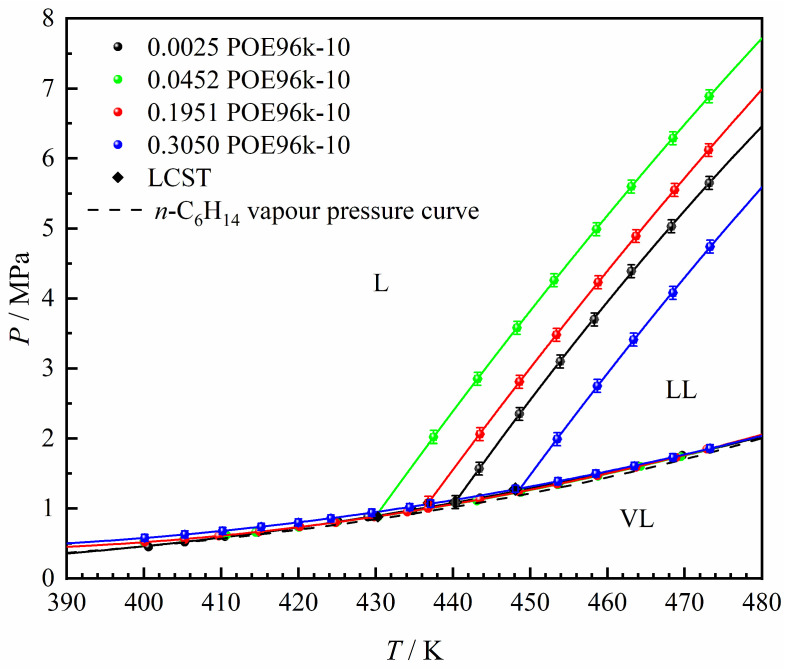
Variation in L → VL, LL → VLL, and L → LL transition pressures (*P*) with temperature (*T*) for the *n*-hexane + POE96k-10 system at different polymer mass fractions. Solid lines represent the results obtained from the modified Sanchez-Lacombe equation of state (MSL EOS).

The phase behavior of the *n*-hexane + POE96k-10 binary system was initially measured as a reference for subsequent studies on the α-olefin + *n*-hexane + POE96k-10 ternary system. The phase equilibrium data for the binary system is provided in Supporting Information Table S2. The data covers a polymer mass fraction range of 0.0025–0.30 g·g^–1^. The polymer concentration range was selected based on conditions relevant to industrial solution processes [39,40,41]. The corresponding *P*–*T* phase diagram was constructed based on the phase behavior data from Table S2, as shown in Figure 3. Specifically, experimental data from the LL and VL lines were separately fitted with quadratic polynomial functions, and their intersection was identified as the LCST point [42,43]. In this work, all LCST points were determined using this method.

The *n*-hexane + POE96k-10 system displays LCST-type phase behavior in Figure 3. This phase behavior is consistent with that typically described for polyolefins in the literature [7,9,15,16,18,20]. In this system, the polymer and solvent form a single liquid phase at lower temperatures, but a liquid–liquid (LL) two-phase region emerges at higher temperatures. Furthermore, Figure 3 shows how the mass fraction of polymer affects the LL phase boundary: as the polymer mass fraction increases, the LL boundary initially shifts toward lower temperatures, but beyond a certain point, it shifts toward higher temperatures.

**Figure 4:**
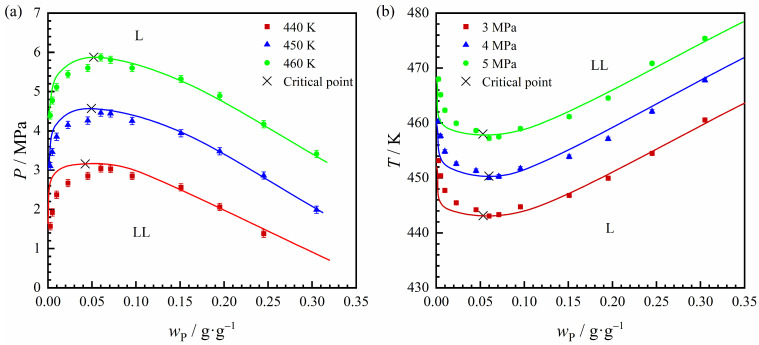
The *P–w*_P_–*T* relationship for the L → LL transition in the *n*-hexane + POE96k-10 system: (**a**) variation in pressure with polymer mass fraction and temperature, and (**b**) variation in temperature with polymer mass fraction and pressure. (×), Critical point; solid lines represent the results obtained from the MSL EOS.

Figure 4 depicts the *P*–*w*_P_–*T* relationship for the L → LL transition in the *n*-hexane + POE96k-10 system. The critical polymer mass fraction is approximately 0.05 g·g^−1^ and appears to remain essentially constant across the experimental temperature and pressure ranges. Figure 4a shows that at a fixed polymer mass fraction, the pressure required for the LL phase transition increases with rising temperature. Meanwhile, Figure 4b indicates that higher pressures result in higher LL phase boundary temperatures.

### 5.2. α-Olefin + Hexane + POE96k-10 Systems

#### 5.2.1. Phase Behavior of the α-Olefin + Hexane + POE96k-10 System

**Figure 5:**
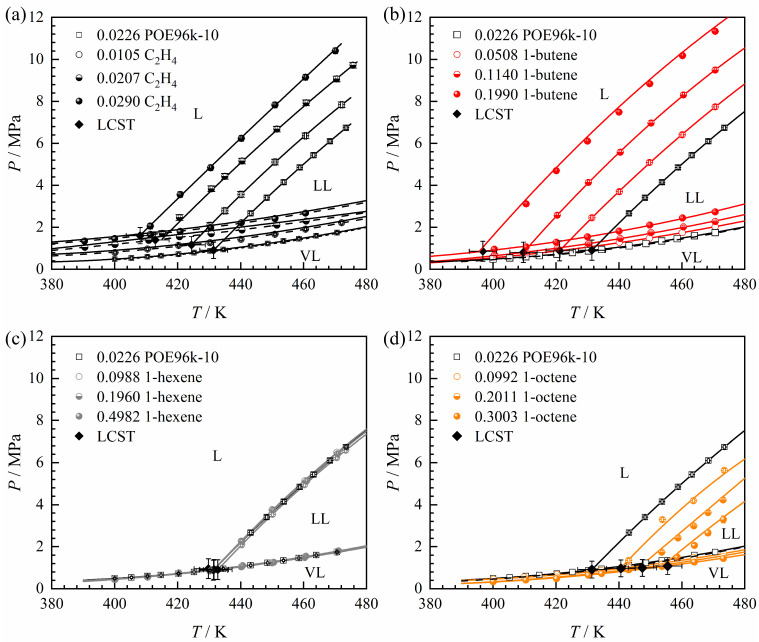
Variation in phase transition pressures with temperature and α-olefin mass fraction for the α-olefin + *n*-hexane + POE96k-10 systems at *w*_P_ ≈ 0.023 g·g^–1^. (**a**) Ethylene + *n*-hexane + POE96k-10 system: the long-dashed line (– – –) represents the vapor-liquid equilibrium pressure of the ethylene + *n*-hexane system; (**b**) 1-butene + *n*-hexane + POE96k-10 system; (**c**) 1-hexene + *n*-hexane + POE96k-10 system; (**d**) 1-octene + *n*-hexane + POE96k-10 system. The short-dashed line (---) represents the vaporization curve of *n*-hexane, and solid lines (─) represent the results obtained from the MSL EOS.

This work examines the phase characteristics of the α-olefin + *n*-hexane + POE96k-10 ternary systems, with α-olefins including C_2_H_4_, 1-C_4_H_8_, 1-C_6_H_12_ and 1-C_8_H_16_ as the third component. Among these α-olefins, C_2_H_4_ is the primary polymerization monomer, while 1-C_4_H_8_, 1-C_6_H_12_ and 1-C_8_H_16_ are widely utilized in industrial POE synthesis as comonomers. The phase transition data for the α-olefin + *n*-hexane + POE96k-10 ternary systems are presented in Table 4, Table 5, Table 6 and Table 7, and the phase boundaries are depicted in Figure 5. The α-olefin content was selected according to industrial production data and is expressed relative to the solvent composition, with the polymer component excluded. To examine the distinct influence of different α-olefins on phase transitions, the *n*-hexane + POE96k-10 system with a polymer mass fraction of 0.0226 g·g^–1^ is used as a reference in this section while keeping the polymer mass fraction of the four ternary systems at approximately 0.023 g·g^–1^.

Figure 5 illustrates that, regardless of the type of α-olefin as the third component, all ternary systems exhibit LCST-type phase behavior, consistent with the binary *n*-hexane + POE96k-10 system. As shown in Figure 5a, ethylene shifts the LL line toward lower temperatures and higher pressures, effectively moving it to the upper left in the phase diagram. The liquid–liquid biphasic region expands, and as the ethylene mass fraction increases, the L → LL phase transition temperature decreases, while the transition pressure increases. The LL phase transition with 1-butene (Figure 5b) is similar to that observed with ethylene. The addition of 1-hexene has a negligible effect on the LL phase transition (Figure 5c). As the 1-hexene mass fraction increases from 0.0988 to 0.4982 g·g^–1^, the liquid–liquid biphasic region sightly expands, with only a minor change. A similar trend is observed for the VL line. In Figure 5d, the addition of 1-octene reduces the size of the liquid–liquid biphasic region, increases the L → LL transition temperature, and decreases the transition pressure. This effect becomes more pronounced as the 1-octene mass fraction increases.

Regarding vapor-liquid phase behavior, the addition of ethylene significantly increases the transition pressures, and 1-butene has a similar effect. Taking experimental uncertainties into account, the vapor-liquid phase transition pressure for the ethylene + *n*-hexane + POE96k-10 system is consistent with the bubble point pressure measured by Nagy et al. [44] for the ethylene + *n*-hexane system, as indicated by the long-dashed line in Figure 5a. This suggests that the polymer has a minimal impact on the L(L) → VL(L) phase transitions. 1-Octene slightly lowers the VL phase transition pressure; however, the change is less pronounced compared to the LL phase transition.

#### 5.2.2. α-Olefin Influence on Phase Transitions

**Figure 6:**
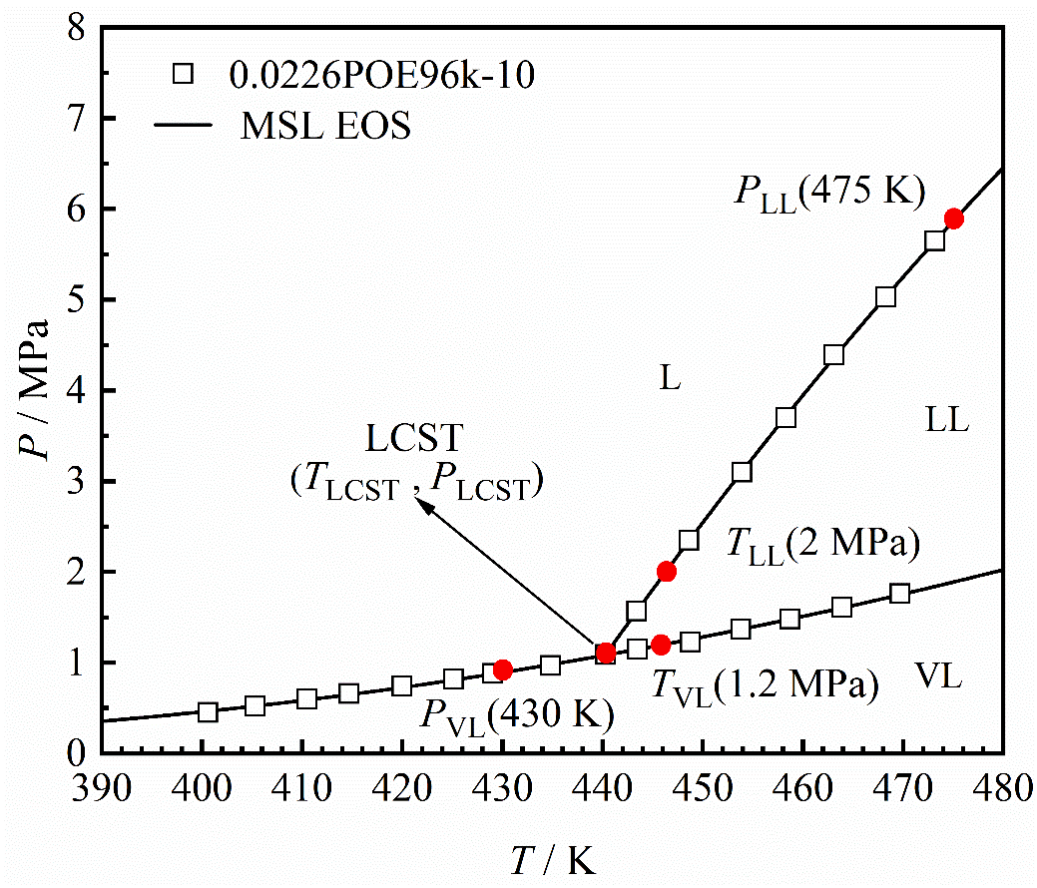
Five key phase transition points in the *P–T* plot for the *n*-hexane + POE96k-10 system.

To quantitatively describe the phase transition of the α-olefin + *n*-hexane + POE96k-10 system, five key phase transition points (corresponding to six phase transition values) were selected from the *P*–*T* phase diagram, as summarized in Figure 6. These five points are the phase transition pressure on the liquid–liquid equilibrium (LLE) line at 475 K, *P*_LL_(475 K); the phase transition temperature on the LLE line at 2 MPa, *T*_LL_(2 MPa); the phase transition pressure on the vapor-liquid equilibrium (VLE) line at 430 K, *P*_VL_(430 K); the phase transition temperature on the VLE line at 1.2 MPa, *T*_VL_(1.2 MPa); and the LCST point (*T*_LCST_, *P*_LCST_) defined as the intersection of the LLE and VLE lines. Among these, *T*_LCST_, *P*_LL_(475 K), and *T*_LL_(2 MPa) are used to analyze the liquid–liquid phase region. The selection of 475 K and 2 MPa is based on the observation that, at these conditions, different α-olefin + *n*-hexane + POE96k-10 systems exhibit cloud point behavior. Alternative temperatures or pressures may also be used, provided that all systems exhibit a cloud point curve under the selected conditions. Similarly, *P*_LCST_, *P*_VL_(430 K), and *T*_VL_(1.2 MPa) are used to analyze the vapor-phase behavior. The selection of 430 K and 1.2 MPa follows the same as above, ensuring that all systems undergo an L(L) → VL(L) phase transitions at these conditions.

**Figure 7:**
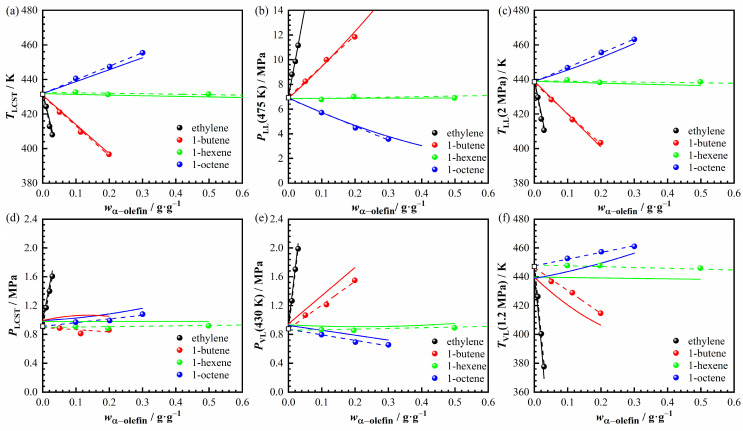
Variation in six phase transition characteristic values with α-olefin content in different α-olefin + *n*-hexane + POE96k-10 systems: (**a**) *T*_LCST_, LCST temperature; (**b**) *P*_LL_(475 K), liquid–liquid transition pressure at 475 K; (**c**) *T*_LL_(2 MPa), liquid–liquid transition temperature at 2 MPa; (**d**) *P*_LCST_, LCST pressure; (**e**) *P*_VL_(430 K), vapor-liquid transition pressure at 430 K; and (**f**) *T*_VL_(1.2 MPa), vapor-liquid transition temperature at 1.2 MPa. The dashed curve (---) illustrates the linear fit of the experimental results, while solid lines (─) correspond to the results derived from the MSL EOS.

Figure 7 presents the variation in the six characteristic phase transition values described above as a function of α-olefin content in different α-olefin + *n*-hexane + POE96k-10 systems. Specifically, Figure 7a–c depict the three characteristic values related to the L → LL transition, while Figure 7d–f represent the three characteristic values associated with L(L) → VL(L) transition. The transition temperature and pressure of L → LL or L(L) → VL(L) phase transitions generally exhibit an approximately linear trend as the α-olefin mass fraction changes. To quantify these trends, linear regressions were performed for the characteristic points and the corresponding slopes, together with the evaluation of confidence intervals. The fitted slopes and their associated 95% confidence intervals are summarized in Table S16. Although the magnitude of the slopes varies among different α-olefins and characteristic points, the statistical analysis confirms that the observed trends are systematically resolvable beyond experimental scatter. In the nonpolar α-olefin + *n*-hexane + POE system, dispersion forces dominate the intermolecular interactions [45]. Due to their quasi-additivity nature [46], variations in α-olefin mass fraction led to proportional changes in the intermolecular interactions between POE and the solvent (*n*-hexane + α-olefin). As a result, the phase transition temperature and pressure also change proportionally as α-olefin mass fraction increases.

**Figure 8:**
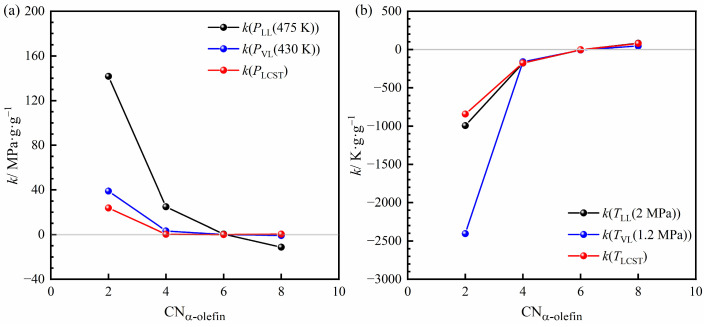
Impact of α-olefin carbon number (CN_α-olefin_) on (**a**) phase transition pressure and (**b**) temperature variation rates with α-olefin mass fraction in α-olefin + *n*-hexane + POE96k-10 systems.

The linear-fit slopes in Figure 7 are listed in Table 8 and further illustrated in Figure 8. These slopes represent the rates of change in phase transition temperature and pressure with respect to the α-olefin mass fraction. As shown in Table 8, when the α-olefin is 1-hexene, the variation rates of all six indicators are close to zero, indicating that 1-hexene has little effect on phase transition temperature and pressure. This behavior can be attributed to the similar molecular chain length of 1-hexene and n-hexane, which leads to comparable dispersion interactions between POE and the two solvents. Additionally, *n*-hexane and 1-hexene have nearly identical vapor pressures, resulting in a negligible influence of 1-hexene on phase behavior, which is consistent with the results in Figure 5c. 1-Butene lowers *T*_LCST_ but has little effect on *P*_LCST_ (Figure 7 and Table 8). Although the decrease in *T*_LCST_ reduces the volatility of the (1-butene + *n*-hexane) mixture at the LCST point, the inherently high volatility of 1-butene increases the L(L) → VL(L) phase transition pressure. These two effects offset each other, resulting in only a minor change in *P*_LCST_.

Figure 8 visually illustrates how different α-olefins influence the variation in phase transition pressure and temperature. Regarding the L → LL phase transition, the variation rates of *T*_LL_(2 MPa) and *T*_LCST_ remain nearly identical (Figure 8b). Meanwhile, for C_4_–C_8_ α-olefins, the variation rate of *P*_LCST_ remains close to zero. The variation rate of *P*_LL_(475 K) shifts from positive for 1-butene to nearly zero for 1-hexene and then to negative for 1-octene. This means *P*_LL_(475 K) increases with 1-butene, remains unchanged with 1-hexene, and decreases with 1-octene. These changes in *P*_LL_(475 K) are primarily influenced by the corresponding shifts in phase transition temperature: the decrease for 1-butene and the increase for 1-octene. Thus, for the L → LL phase transition, C_4_–C_8_ α-olefins mainly affect phase transition temperature rather than pressure.

The addition of 1-butene lowers the L → LL phase transition temperature, while 1-hexene has little effect. In contrast, 1-octene increases the phase transition temperature (Figure 5 and Figure 8). Since α-olefins share a similar chemical structure with *n*-alkanes, their intermolecular interactions resemble those of *n*-alkanes with the same carbon number. Moreover, the dispersion force involving the polymer and α-olefin generally increases with the molar mass of the α-olefin. As a result, the dispersion force involving the polymer and 1-butene is weaker than that with *n*-hexane, whereas for 1-hexene, it remains similar to that with *n*-hexane. In contrast, the dispersion force between the polymer and 1-octene is stronger. This phenomenon can be explained by the thermodynamic criterion for homogeneous solution formation from multiple pure components:

(9) $$\Delta G_{m}=\Delta H_{m}-T\Delta S_{m}<0$$

According to this equation, temperature correlates with Δ*H*_m_/Δ*S*_m_. Since phase separation occurs upon heating in LCST-type phase behavior, Δ*S*_m_ < 0 [47]. Stronger dispersion forces make mixing more exothermic, leading to a more negative Δ*H*_m_ and, consequently, a higher phase transition temperature. Conversely, weaker dispersion forces between the polymer and solvent result in a lower L → LL transition temperature.

As shown in Figure 8, when the α-olefin is 1-hexene or 1-octene, their effects on the VL phase transition pressure are nearly identical. Although the vapor pressures of 1-hexene and 1-octene differ, the VL phase transition is still predominantly governed by n-hexane. In contrast, α-olefins with higher volatility than n-hexane, such as ethylene or 1-butene, exert a significant influence on the VL phase transition pressure. This influence becomes more pronounced as the α-olefin carbon number decreases.

Compared to other α-olefins, ethylene exhibits the most pronounced influence on both phase-transition temperature and pressure. While dispersion interactions generally increase with the molar mass of α-olefins, ethylene shows a clear deviation from this trend and behaves as a strong anti-solvent. This behavior cannot be explained by dispersion forces alone and suggests a reduced effective affinity between ethylene and the polymer–solvent system. Unlike *n*-alkanes, α-olefins contain a carbon–carbon double bond, which may introduce additional electronic effects. For ethylene, owing to its short molecular chain, such effects may become relatively more significant compared to dispersive interactions. From a thermodynamic perspective, this behavior can be qualitatively rationalized in terms of a weaker effective polymer–solvent affinity, which reduces polymer solvation and shifts the L → LL phase boundary to lower temperatures. In the absence of direct quantitative thermodynamic analysis, this interpretation should be regarded as a plausible qualitative explanation rather than a definitive mechanism. Additionally, ethylene significantly increases *P*_LCST_ and *P*_VL_(430 K) in Figure 8a. This is because ethylene has much higher volatility than *n*-hexane, making the L(L) → VL(L) phase behavior primarily controlled by ethylene. Ethylene simultaneously reduces the transition temperature and markedly raises the transition pressure.

### 5.3. Modeling Results

The method for obtaining individual component parameters in Section 3 of the MSL EOS is described and summarized in Table S1. Solvent–solvent binary interaction parameters are determined through correlation with phase equilibrium data from the literature. Polymer–solvent binary interaction parameters are modeled as linear functions of temperature to improve cloud point predictions. Both $k_{ij}^{a}$ and $k_{ij}^{b}$ parameters are fitted simultaneously. The Generalized Reduced Gradient method minimizes the objective function (OF) to determine optimal *k_ij_* values:

(10) $$\mathrm{OF}=\sqrt{\sum_{i=1}^{N}{\left(\frac{P_{i,\mathrm{calc}}-P_{i,\exp}}{P_{i,\exp}}\right)}^{2}}$$

The binary interaction parameters for *n*-hexane–POE96k-10 are obtained by correlating all L → LL phase equilibrium data. Similarly, the parameters for α-olefin–POE96k-10 are derived from the correlation of L → LL phase equilibrium data (Table 4, Table 5, Table 6 and Table 7). For C_2_H_4_, 1-C_4_H_8_, 1-C_6_H_12_ and 1-C_8_H_16_, the LL data at mass fractions of 0.0207, 0.1140, 0.1960, and 0.2011 g·g^–1^, respectively, were selected for correlation. All the binary interaction parameters employed in this work are provided in Table 9. Figure 3 presents the MSL EOS correlation results for the *n*-hexane + POE96k-10 phase behavior, while Figure 5a–d show both the correlation and prediction results for the α-olefin + *n*-hexane + POE96k-10 system. Table 10 reports the average absolute deviation (AAD) between experimental values and those calculated using the MSL EOS for the phase behavior of the binary and ternary systems.

Table 10 shows that the MSL EOS exhibits a good correlation for the *n*-hexane + POE96k-10 system, with an AAD(*P*_LL_) of 0.14 MPa. It accurately describes the dependence of L → LL pressure on temperature and polymer mass fraction. For the α-olefin + *n*-hexane + POE96k-10 system, the MSL EOS shows excellent correlation when the α-olefin is ethylene or 1-butene, with an AAD below 0.08 MPa. The correlation remains satisfactory for 1-hexene. However, for 1-octene, the AAD(*P*_LL_) increases to 0.45 MPa, indicating reduced accuracy. In summary, the correlation accuracy decreases as the α-olefin carbon chain length increases. The binary interaction parameters in Table 10 can effectively predict the LL and VL phase transition pressures for the α-olefin + *n*-hexane + POE96k-10 system. The prediction of VL phase behavior is more accurate than that of the LL phase transition pressure, as AAD(*P*_VL_) is generally smaller than AAD(*P*_LL_). Additionally, as shown in Figure 7, the MSL EOS accurately reproduces the L → LL phase transition pressure at low α-olefin mass fractions. However, the discrepancy between the predicted and actual values increases with α-olefin mass fraction. Notably, only in the ethylene + *n*-hexane + POE96k-10 system does the MSL EOS accurately relate the L → LL phase transition pressure at high ethylene mass fractions.

### 5.4. Design Implications for Solution Polymerization

Although the present study primarily focuses on the fundamental phase behavior of polymer–solvent systems, the obtained characteristic phase-transition data can be directly translated into operating guidelines for solution polymerization processes. In particular, the experimentally determined L → LL phase boundaries and LCST points provide useful constraints for defining safe single-phase operating windows.

Under typical polymer concentrations encountered in industrial solution polymerization, liquid–liquid (LL) phase separation is undesirable, as it may lead to polymer precipitation, catalyst deactivation, or operational instability. Based on the measured phase-boundary data, maintaining operating pressures above the L → LL transition boundary at a given temperature is required to ensure homogeneous conditions. Accordingly, the LCST point may be regarded as a conservative lower limit for pressure selection at a given operating temperature.

The LL and VL phase-transition temperature and pressure vary approximately linearly with α-olefin mass fraction, enabling an estimation of operating limits as a function of comonomer concentration. From a process perspective, C_2_H_4_, 1-C_4_H_8_ lower the LL transition temperatures and narrow the single-phase operating window, whereas 1-C_8_H_16_ increases the transition temperature and expands this window. Ethylene exhibits a strong anti-solvent effect, significantly increasing the LL transition pressure due to its high volatility, and therefore requires higher operating pressures to suppress LL phase separation.

## 6. Conclusions

Building on phase equilibrium data measurements of the *n*-hexane + POE96k-10 binary system, this study further investigates the phase transition behavior of the α-olefin + *n*-hexane + POE96k-10 ternary system. The influences of C_2_H_4_, 1-C_4_H_8_, 1-C_6_H_12_ and 1-C_8_H_16_ on the solvent + POE system are analyzed. The MSL EOS is employed to correlate and reproduce phase equilibrium based on the solvent–POE96k-10 binary interaction parameters. The results show that the liquid–liquid (LL) and vapor–liquid (VL) phase-transition temperature and pressure vary approximately linearly with α-olefin mass fraction, which is explained by the additivity principle of dispersion forces. Different α-olefins influence phase behavior differently: C_2_H_4_, 1-C_4_H_8_ and 1-C_6_H_12_ lower the LL and VL phase transition temperature, whereas 1-C_8_H_16_ raises it. Additionally, ethylene acts a strong anti-solvent, likely due to its π-electron repulsion, which weakens polymer-solvent interactions. Notably, unlike other α-olefins that primarily affect phase transition temperature, ethylene not only lowers the temperature but also significantly increases the pressure, primarily due to its high volatility. The MSL EOS provides a consistent description of the phase behavior of both the *n*-hexane + POE96k-10 binary system and the α-olefin + *n*-hexane + POE96k-10 ternary system. However, its accuracy declines with increasing α-olefin chain length. Interaction parameters derived from L → LL phase transition correlations at moderate α-olefin mass fractions capture the observed phase behavior at low α-olefin concentrations, while increasing deviations are observed at higher concentrations.

## Figures and Tables

**Table 1:** Summary of the chemicals employed in this research.

Name	CAS Number	Supplier	Purity/% *^a^*
nitrogen	7727-37-9	Shuntai Gas	99.99
ethylene	74-85-1	Shuntai Gas	99.9
1-butene	106-98-9	Shuntai Gas	≥99
1-hexene	592-41-6	Aladdin Scientific	99.67
1-octene	111-66-0	Aladdin Scientific	99.72
*n*-hexane	110-54-3	Aladdin Scientific	98.52
xylene	1330-20-7	Aladdin Scientific	≥98.5 *^b^*

*^a^* The purity of the chemicals was provided by the supplier. *^b^* Xylene is a mixture of ortho-, meta-, para-xylene, as well as ethylbenzene.

**Table 2:** Characterization results of the ethylene/1-octene copolymer (POE96k-10).

Polymer Characteristic	Value
Molecular Weight and Distribution	
*M_w_*/g·mol^–1^	95,900
*M_n_*/g·mol^–1^	24,800
*M_z_*/g·mol^–1^	172,500
*M_w_*/*M_n_*	3.87
Comonomer Content	
1-octene mole fraction, mol%	10.31
1-octene mass fraction, wt%	31.50
branching density, SCB/1000TC	69.50

**Table 3:** Distribution of 17 pseudocomponents for POE96k-10.

i	M_i_/g·mol^–1^	w_i_/g·g^–1^	i	M_i_/g·mol^–1^	w_i_/g·g^–1^
1	1.1379 × 10^2^	2.2571 × 10^–5^	11	2.4698 × 10^4^	9.9589 × 10^–2^
2	1.6916 × 10^2^	5.1006 × 10^–4^	12	4.3307 × 10^4^	2.0544 × 10^–1^
3	2.9200 × 10^2^	1.7686 × 10^–3^	13	7.5496 × 10^4^	2.7800 × 10^–1^
4	4.8688 × 10^2^	2.1582 × 10^–3^	14	1.3373 × 10^5^	2.2092 × 10^–1^
5	8.6861 × 10^2^	1.8285 × 10^–3^	15	2.3999 × 10^5^	9.9279 × 10^–2^
6	1.5329 × 10^3^	2.0383 × 10^–3^	16	4.4887 × 10^5^	2.0868 × 10^–2^
7	2.6959 × 10^3^	3.1374 × 10^–3^	17	1.2311 × 10^6^	4.7116 × 10^–6^
8	4.7289 × 10^3^	6.5647 × 10^–3^			
9	8.1729 × 10^3^	1.6567 × 10^–2^			
10	1.4138 × 10^4^	4.1296 × 10^–2^			
*M*_n_/g·mol^–1^		25,505			
*M*_w_/g·mol^–1^		95,853			
*M*_z_/g·mol^–1^		166,100			

**Table 4:** Phase transition pressures for the ethylene + *n*-hexane + POE96k-10 system at various polymer mass fractions, temperatures and ethylene mass fractions *^d^*.

*w*_p_ = 0.0226 g·g^–1^			*w*_p_ = 0.0228 g·g^–1^			*w*_p_ = 0.0226 g·g^–1^		
*w*(C_2_H_4_) = 0.0105 g·g^–1^ *^b^*			*w*(C_2_H_4_) = 0.0207 g·g^–1 *b*^			*w*(C_2_H_4_) = 0.0290 g·g^–1 *b*^		
*T* (K)	*P* (MPa)	Phase *^a^*	*T* (K)	*P* (MPa)	Phase *^a^*	*T* (K)	*P* (MPa)	Phase *^a^*
400.1	0.80	VL	390.5	1.05	VL	380	1.22	VL
410.4	0.97	VL	400.1	1.18	VL	390.4	1.35	VL
420.3	1.14	VL	410.8	1.38	VL	400	1.47	VL
430.3	1.27	VL	420.6	1.55	VL	411.3	1.67	VL
440.1	1.43	VL	430.7	1.70	VL	420.8	1.81	VL
450.9	1.67	VL	440.4	1.87	VL	430.5	1.99	VL
460.6	1.91	VL	451.5	2.10	VL	440.2	2.22	VL
472.1	2.22	VL	460.8	2.30	VL	450.9	2.42	VL
424.4	1.17	LCST *^c^*	412.8	1.40	LCST *^c^*	460.6	2.68	VL
430.3	2.09	LL	415.1	1.71	LL	408.1	1.61	LCST *^c^*
435	2.79	LL	420.6	2.47	LL	411.3	2.06	LL
440.1	3.57	LL	430.7	3.84	LL	420.8	3.56	LL
450.9	5.11	LL	435	4.43	LL	430.5	4.84	LL
460.6	6.36	LL	440.4	5.16	LL	440.2	6.24	LL
472.1	7.84	LL	451.5	6.68	LL	450.9	7.83	LL
			460.8	7.93	LL	460.6	9.15	LL
			470.5	9.07	LL	470.1	10.41	LL
			475.7	9.72	LL			

*^a^* VL denotes the vapor-liquid phase transition, and LL denotes the liquid–liquid phase transition; *^b^* The mass fraction of ethylene was calculated based on the solvent only, excluding the polymer; *^c^* The lower critical solution temperature (LCST) point was determined as the intersection of polynomial fits to the LL and VL phase-boundary data. *^d^* The combined standard uncertainties are *u*_c_(*w_P_*) = 0.0005 g·g^–1^, *u*_c_(*w*_ethylene_) = 0.0008 g·g^–1^, *u*_c_(*T*) = 0.35 K, *u*_c_(*P*) = 0.058 MPa for the L → VL and LL → VLL transitions, and *u*_c_(*P*) = 0.135 MPa for the L → LL transition.

**Table 5:** Phase transition pressures for the 1-butene + *n*-hexane + POE96k-10 system at various polymer mass fractions, temperatures and 1-butene mass fractions *^d^*.

*w*_p_ = 0.0230 g·g^–1^			*w*_p_ = 0.0231 g·g^–1^			*w*_p_ = 0.0233 g·g^–1^		
*w*(1-butene) = 0.0508 g·g^–1 *b*^			*w*(1-butene) = 0.1140 g·g^–1 *b*^			*w*(1-butene) = 0.1990 g·g^–1 *b*^		
*T* (K)	*P* (MPa)	Phase *^a^*	*T* (K)	*P* (MPa)	Phase *^a^*	*T* (K)	*P* (MPa)	Phase *^a^*
400	0.58	VL	400.5	0.69	VL	400.4	0.96	VL
410.3	0.65	VL	410.7	0.78	VL	410.5	1.07	VL
420.3	0.88	VL	420.4	1.00	VL	420.1	1.29	VL
430.2	1.07	VL	430.3	1.22	VL	430	1.55	VL
440.1	1.29	VL	440.4	1.47	VL	440	1.82	VL
449.7	1.48	VL	450.2	1.75	VL	449.8	2.11	VL
460.1	1.75	VL	460.5	2.02	VL	460.2	2.45	VL
470.6	2.03	VL	470.6	2.28	VL	470.6	2.74	VL
421.1	0.88	LCST *^c^*	409.6	0.81	LCST *^c^*	396.7	0.86	LCST *^c^*
430.2	2.27	LL	410.5	0.96	LL	400.4	1.47	LL
431.4	2.46	LL	420.4	2.58	LL	410.5	3.12	LL
440.1	3.70	LL	430.3	4.14	LL	420.1	4.70	LL
449.7	5.09	LL	440.4	5.58	LL	430	6.11	LL
460.1	6.41	LL	450.2	6.97	LL	440	7.49	LL
470.6	7.74	LL	460.5	8.31	LL	449.8	8.84	LL
475.5	8.31	LL	470.6	9.49	LL	460.2	10.18	LL
			475.7	10.08	LL	470.6	11.34	LL
						475.9	11.94	LL

*^a^* VL denotes the vapor-liquid phase transition, and LL denotes the liquid–liquid phase transition; *^b^* The mass fraction of 1-butene was calculated based on the solvent only, excluding the polymer; *^c^* The LCST point was determined as the intersection of polynomial fits to the LL and VL phase-boundary data. *^d^* The combined standard uncertainties are *u*_c_(*w_P_*) = 0.0004 g·g^–1^, *u*_c_(*w*_1-butene_) = 0.0009 g·g^–1^, *u*_c_(*T*) = 0.35 K, *u*_c_(*P*) = 0.051 MPa for the L → VL and LL → VLL transitions, and *u*_c_(*P*) = 0.113 MPa for the L → LL transition.

**Table 6:** Phase transition pressures for the 1-hexene + *n*-hexane + POE96k-10 system at various polymer mass fractions, temperatures and 1-butene mass fractions *^d^*.

*w*_p_ = 0.0220 g·g^–1^			*w*_p_ = 0.0213 g·g^–1^			*w*_p_ = 0.0234 g·g^–1^		
*w*(1-hexene) = 0.0988 g·g^–1 *b*^			*w*(1-hexene) = 0.1960 g·g^–1 *b*^			*w*(1-hexene) = 0.4982 g·g^–1 *b*^		
*T* (K)	*P* (MPa)	Phase *^a^*	*T* (K)	*P* (MPa)	Phase *^a^*	*T* (K)	*P* (MPa)	Phase *^a^*
400.1	0.43	VL	400	0.46	VL	400.1	0.48	VL
410.4	0.58	VL	409.9	0.56	VL	410.5	0.55	VL
421.1	0.71	VL	420.2	0.73	VL	419.8	0.76	VL
431.2	0.86	VL	430.2	0.85	VL	430.5	0.91	VL
440.3	1.05	VL	440.2	1.04	VL	440.6	1.10	VL
450	1.23	VL	450	1.23	VL	450.5	1.26	VL
460.3	1.48	VL	460.4	1.51	VL	460.7	1.55	VL
470.5	1.73	VL	470.6	1.77	VL	471.1	1.80	VL
432.7	0.91	LCST *^c^*	431.3	0.88	LCST *^c^*	431.5	0.92	LCST *^c^*
440.3	2.09	LL	440.2	2.27	LL	440.4	2.26	LL
450.1	3.54	LL	450	3.77	LL	450.3	3.75	LL
460.3	4.95	LL	460.4	5.14	LL	461	5.14	LL
470.5	6.24	LL	470.6	6.49	LL	470.8	6.42	LL
473.4	6.59	LL	475.7	7.09	LL	475.3	6.95	LL
475.8	6.87	LL						

*^a^* VL denotes the vapor-liquid phase transition, and LL denotes the liquid–liquid phase transition; *^b^* The mass fraction of 1-hexene was calculated based on the solvent only, excluding the polymer; *^c^* The LCST point was determined as the intersection of polynomial fits to the LL and VL phase behavior data. *^d^* The combined standard uncertainties are *u*_c_(*w_P_*) = 0.0004 g·g^–1^, *u*_c_(*w*_1-hexene_) = 0.0016 g·g^–1^, *u*_c_(*T*) = 0.35 K, *u*_c_(*P*) = 0.051 MPa for the L → VL and LL → VLL transitions, and *u*_c_(*P*) = 0.092 MPa for the L → LL transition.

**Table 7:** Phase transition pressures for the 1-octene + *n*-hexane + POE96k-10 system at various polymer mass fractions, temperatures and 1-octene mass fractions *^d^*.

*w*_p_ = 0.0230 g·g^–1^			*w*_p_ = 0.0228 g·g^–1^			*w*_p_ = 0.0231 g·g^–1^		
*w*(1-octene) = 0.0992 g·g^–1 *b*^			*w*(1-octene) = 0.2011 g·g^–1 *b*^			*w*(1-octene) = 0.3003 g·g^–1 *b*^		
*T* (K)	*P* (MPa)	Phase *^a^*	*T* (K)	*P* (MPa)	Phase *^a^*	*T* (K)	*P* (MPa)	Phase *^a^*
400.1	0.45	VL	400	0.33	VL	400.1	0.34	VL
409.9	0.55	VL	410.4	0.45	VL	410.4	0.41	VL
420.3	0.67	VL	420.3	0.55	VL	420.2	0.48	VL
430.6	0.81	VL	430.2	0.68	VL	430.3	0.64	VL
443.1	1.01	VL	443.2	0.91	VL	443.1	0.90	VL
453.8	1.18	VL	453.6	1.15	VL	453.7	1.06	VL
463.8	1.48	VL	463.8	1.39	VL	464.0	1.27	VL
473.2	1.67	VL	473.1	1.53	VL	473.2	1.44	VL
440.6	0.97	LCST *^c^*	447.4	0.99	LCST *^c^*	455.4	1.08	LCST *^c^*
443.1	1.35	LL	453.6	1.74	LL	458.5	1.50	LL
453.8	3.62	LL	458.8	2.42	LL	464.0	2.06	LL
463.7	4.19	LL	463.8	2.99	LL	468.3	2.66	LL
473.2	5.48	LL	473.1	4.23	LL	473.2	3.30	LL
475.7	5.51	LL	475.4	4.53	LL	475.4	3.61	LL

*^a^* VL denotes the vapor-liquid phase transition, and LL denotes the liquid–liquid phase transition; *^b^* The mass fraction of 1-octene was calculated based on the solvent only, excluding the polymer; *^c^* The LCST point was determined as the intersection of polynomial fits to the LL and VL phase behavior data. *^d^* The combined standard uncertainties are *u*_c_(*w_P_*) = 0.0004 g·g^–1^, *u*_c_(*w*_1-hexene_) = 0.0022 g·g^–1^, *u*_c_(*T*) = 0.35 K, *u*_c_(*P*) = 0.051 MPa for the L → VL and LL → VLL transitions, and *u*_c_(*P*) = 0.092 MPa for the L → LL transition.

**Table 8:** Variation rates of phase transition pressure and temperature with α-olefin mass fraction in different α-olefin + *n*-hexane + POE96k-10 systems, obtained through linear fitting of the experimental data in Figure 7.

*k*	C_2_H_4_	1-C_4_H_8_	1-C_6_H_12_	1-C_8_H_16_
*k*(*T*_LCST_)/K·g·g^–1^	–841.9	–173.5	–2.211	78.74
*k*(*P*_LL_(475 K))/MPa·g·g^–1^	141.8	24.9	0.451	–11.24
*k*(*T*_LL_(2 MPa))/K·g·g^–1^	–993.5	–176.2	–2.41	82.36
*k*(*P*_LCST_)/MPa·g·g^–1^	23.74	–0.317	0.055	0.518
*k*(*P*_VL_(430 K))/MPa·g·g^–1^	38.9	3.3	0.092	–0.76
*k*(*T*_VL_(1.2 MPa))/K·g·g^–1^	–2404	–159	–5.79	46.5

**Table 9:** Binary interaction parameters of the MSL EOS.

*j*	*n*-C_6_H_14_	POE96k-10	
	${k_{ij}}^{\;a}$	$k_{ij}^{a}$	$k_{ij}^{b}$/K^−1^
*n*-C_6_H_14_	/	–1.524 × 10^–1^	3.477 × 10^–4^
C_2_H_4_	2.905 × 10^–2^	–3.265 × 10^–4^	1.726 × 10^–4^
1-C_4_H_8_	7.800 × 10^–3^	5.284 × 10^–5^	–2.155 × 10^–6^
1-C_6_H_12_	5.753 × 10^–3^	–3.883 × 10^–5^	–6.097 × 10^–5^
1-C_8_H_16_	2.082 × 10^–3^	0.924 × 10^–1^	–2.064 × 10^–4^

*^a^* Taken from ref. [37].

**Table 10:** AAD between experimental values and those calculated for the phase behavior of the binary and ternary systems using the MSL EOS.

α-Olefin	Correlation *^a^*	Prediction *^b^*	
	AAD(*P*_LL_)/MPa	AAD(*P*_VL_)/MPa	AAD(*P*_LL_)/MPa
/	0.14	0.05	/
C_2_H_4_	0.07	0.10	0.10
1-C_4_H_8_	0.08	0.10	0.25
1-C_6_H_12_	0.12	0.12	0.26
1-C_8_H_16_	0.45	0.13	0.31

*^a^* For the *n*-hexane + POE96k-10 system, all cloud point data were used to correlate the MSL EOS, whereas for the α-olefin + *n*-hexane + POE96k-10 system, only cloud point data at intermediate α-olefin mass fractions were included in the correlation. *^b^* Data used for correlation were excluded when calculating AAD between predicted and experimental data.

## Data Availability

The relevant data generated and analyzed in the current study are available from the corresponding author upon reasonable request.

## Supplementary Materials

The following supporting information can be downloaded at https://www.mdpi.com/article/10.3390/polym18010064/s1, Table S1: Physical properties and MSL equation parameters for each component; Table S2: Phase transition pressures for the n-hexane + POE96k-10 system at different temperatures and polymer concentrations. Table S3. Methods for determining standard uncertainty from non-statistical definitions. Table S4. Dependence of the transition pressures *P* on the temperature *T*, polymer mass fraction *w*_P_, and α-olefin mass fraction *w*_α-olefin_. Table S5. Summary of standard uncertainties *u*_c_ of the reported temperatures, pressures, polymer mass fractions *w*_P_, and α-olefin mass fractions *w*_α-olefin_. Table S6. Illustrative polynomial coefficients (mean ± standard error) used in the Monte Carlo example. Table S7. Uncertainty and 95% confidence intervals of LCST temperature and pressure for different (α-olefin + n-hexane + POE96k-10) systems. Table S8. Quadratic phase-boundary fitting parameters (mean ± standard error) for the determination of LCST in the (n-hexane + POE96k-10) and (α-olefin + n-hexane + POE96k-10) systems. Table S9. Detailed uncertainty analysis for phase transition temperature. Table S10. Detailed uncertainty analysis for pressure measurement. Table S11. Detailed uncertainty analysis of the polymer loading mass. Table S12. Detailed Uncertainty Report for the Composition of Solvent Mixture Loaded. Table S13. Detailed Uncertainty Report for the Mass of Solvent Mixture Loaded. Table S14. Detailed Uncertainty Report for the Mass of Gas (ethylene or 1-butene) Loaded. Table S15. Comparison of LCST temperature and pressure obtained from quadratic and cubic polynomial fits for different solvent systems. Table S16. Linear regression slopes of characteristic points for different α-olefin + n-hexane + POE96k-10 systems.
