# Supplementary material for: High-Pressure Phase Behavior of α-Olefin + n-Hexane + Ethylene/1-Octene Copolymer Systems: Experimental Study and Modeling
Source: Polymers (Basel). 2025 Dec 25;18(1):64. doi: 10.3390/polym18010064 (PMC12787770; doi:10.3390/polym18010064)
Supplement: Supplementary file 1 [file polymers-18-00064-s001.zip › polymers-4038074-supplementary.pdf]

High-Pressure Phase Behavior of α-Olefin + n-Hexane + Ethylene/1-Octene Copolymer Systems: Experimental Study and Modeling

Ruijun Zhang [1], Ziyi Dong [2], Qiqi He [1], Junhua Li [2], Yuexin Hu [2] and Jianhua Qian [2,*]

[1] College of Chemistry and Chemical Engineering, China University of Petroleum (East China),
Qingdao 266555, China; zhangruijun523@gmail.com (R.Z.); qqhe65878@gmail.com (Q.H.)

[2] College of Petrochemical Engineering, Liaoning Petrochemical University, Fushun 113001, China; 13898210007@163.com (Z.D.); lijunhua0521@163.com (J.L.); yxhu1981@163.com (Y.H.)

* Correspondence: qianjh@lnpu.edu.cn

S.1 Supplementary Data

Table S1. Physical properties [a] and MSL equation parameters [b] for each component.

	C_2H_4	1-C_4H_8	1-C_6H_{12}	1-C_8H_{16}	n-C_6H_{14}	POE96k-10 [c]
M/g·mol^{-1}	28.05	56.11	84.16	112.21	86.17	
T_c/K	282.34	419.5	504	566.9	507.6	
P_c/MPa	5.041	4.02	3.21	2.663	3.025	
V_c/cm^3·mol^{-1}	131.1	241	348	464	371	
ω	0.0870	0.1845	0.2851	0.3921	0.3013	
ε/kJ·mol^{-1}	2.291	3.215	3.681	3.97	3.682	4.014
v*/cm^3·mol^{-1}	7.516	10.470	12.092	12.769	12.422	8.411
(d/M)/mol·g^{-1}	0.22609	0.13930	0.11216	0.10048	0.11267	0.06986
c/cm^3·g^{-1}	0.6362	0.3501	0.2606	0.2221	0.2623	–0.5059

[a] T_c, critical temperature; P_c, critical pressure; V_c, critical volume; ω, acentric factor. [b] ε, lattice energy; v*, volume per lattice site; d, number of lattice sites; c, volume shift factor. [c] Taken from ref [37].

Table S2. Phase transition pressures for the n-hexane + POE96k-10 system at different temperatures and polymer concentrations. [d]

T(K)	P(MPa)	Phase [a]	T(K)	P(MPa)	Phase [a]	T(K)	P(MPa)	Phase [a]
w_p = 0.0025g·g^{-1}			w_p = 0.0050g·g^{-1}			w_p = 0.0098g·g^{-1} [c]		
400.6	0.45	VL	400.1	0.49	VL	443.4	2.37	LL
405.3	0.52	VL	405.2	0.56	VL	448.2	3.07	LL
410.5	0.60	VL	410.5	0.63	VL	453.8	3.85	LL
414.7	0.66	VL	414.9	0.69	VL	458.5	4.51	LL
420	0.74	VL	420.2	0.77	VL	463.1	5.11	LL
425.1	0.82	VL	425.0	0.83	VL	468.5	5.79	LL
429	0.88	VL	430.1	0.90	VL	473	6.35	LL
434.8	0.97	VL	434.6	0.96	VL			
443.5	1.15	VL	443.4	1.14	VL			
448.8	1.23	VL	448.9	1.25	VL			
453.8	1.37	VL	453.7	1.35	VL			
463.9	1.61	VL	458.8	1.47	VL			
469.7	1.76	VL	463.9	1.60	VL			
440.3	1.09	LCST [b]	469.7	1.75	VL			
443.4	1.57	LL	436.9	1.02	LCST [b]			
448.6	2.35	LL	442.9	1.92	LL			
453.9	3.10	LL	448.4	2.73	LL			
458.3	3.70	LL	453.6	3.46	LL			
463.1	4.39	LL	458.4	4.10	LL			
468.3	5.03	LL	463.3	4.77	LL			
473.2	5.65	LL	468.5	5.44	LL			

			473.1	6.01	LL			
$w_p = 0.0226\text{g}\cdot\text{g}^{-1}$			$w_p = 0.0452\text{g}\cdot\text{g}^{-1}$			$w_p = 0.0600\text{g}\cdot\text{g}^{-1}$ [c]		
400	0.49	VL	40	0.52	VL	437.7	2.21	LL
405.3	0.54	VL	405.2	0.56	VL	443.4	3.04	LL
410.4	0.60	VL	410.7	0.62	VL	448.5	3.78	LL
414.7	0.65	VL	414.5	0.66	VL	453.3	4.46	LL
420.3	0.72	VL	420.1	0.73	VL	458.6	5.19	LL
425.2	0.80	VL	424.9	0.80	VL	463.8	5.87	LL
430.3	0.88	VL	430.3	0.88	VL	468.3	6.45	LL
434.5	0.95	VL	434.1	0.95	VL	473.1	7.06	LL
443.2	1.12	VL	443.1	1.11	VL			
448.6	1.23	VL	448.8	1.23	VL			
453.6	1.35	VL	453.6	1.34	VL			
458.5	1.46	VL	458.8	1.46	VL			
464	1.6	VL	464.3	1.60	VL			
470.6	1.75	VL	469.5	1.74	VL			
431.4	0.91	LCST [b]	429.9	0.88	LCST [b]			
443.2	2.67	LL	437.5	2.02	LL			
448.3	3.41	LL	443.2	2.85	LL			
453.7	4.15	LL	448.3	3.58	LL			
458.8	4.85	LL	453.1	4.26	LL			
463.2	5.44	LL	458.6	4.99	LL			
468.4	6.1	LL	463.1	5.60	LL			
			468.5	6.29	LL			
			473.2	6.89	LL			
$w_p = 0.0710\text{g}\cdot\text{g}^{-1}$ [c]			$w_p = 0.0955\text{g}\cdot\text{g}^{-1}$			$w_p = 0.1510\text{g}\cdot\text{g}^{-1}$ [c]		
437.0	2.06	LL	400.1	0.51	VL	437.2	1.57	LL
443.5	3.03	LL	405.5	0.55	VL	443.8	2.56	LL
448.3	3.72	LL	410.5	0.60	VL	448.3	3.21	LL
453.4	4.44	LL	414.8	0.65	VL	453.4	3.94	LL
458.6	5.15	LL	420.2	0.72	VL	458.5	4.64	LL
463.6	5.81	LL	424.9	0.79	VL	463.6	5.32	LL
468.4	6.42	LL	430.3	0.87	VL	468.4	5.95	LL
473.1	7.02	LL	436.1	0.97	VL	473	6.56	LL
			443.6	1.11	VL			
			448.7	1.21	VL			
			453.3	1.32	VL			
			458.1	1.43	VL			
			463.1	1.55	VL			
			468	1.68	VL			

			472.2	1.79	VL			
			430.5	0.89	LCST [b]			
			437.2	1.89	LL			
			443.6	2.85	LL			
			448.2	3.50	LL			
			453.5	4.25	LL			
			458.4	4.92	LL			
			463.5	5.60	LL			
			468.2	6.22	LL			
			472.8	6.80	LL			
$w_p = 0.1951 g\cdot g^{-1}$			$w_p = 0.2450 g\cdot g^{-1}$			$w_p = 0.3050 g\cdot g^{-1}$		
400.1	0.52	VL	400	0.53	VL	400.1	0.58	VL
405.3	0.56	VL	405.2	0.57	VL	405.3	0.63	VL
409.7	0.61	VL	410.1	0.62	VL	410.2	0.68	VL
415	0.67	VL	414.9	0.68	VL	415.2	0.74	VL
420.2	0.74	VL	420.9	0.75	VL	420.0	0.80	VL
424.8	0.80	VL	424.4	0.80	VL	424.2	0.86	VL
429.8	0.88	VL	429.5	0.88	VL	429.5	0.94	VL
434.1	0.95	VL	434	0.95	VL	434.4	1.02	VL
436.8	1.00	VL	436.9	1.00	VL	437.1	1.07	VL
443.5	1.13	VL	443.6	1.13	VL	448.1	1.28	VL
448.5	1.24	VL	448.3	1.25	VL	453.6	1.39	VL
453.5	1.35	VL	453.8	1.34	VL	458.5	1.50	VL
458.7	1.47	VL	458.1	1.44	VL	463.5	1.61	VL
463.7	1.59	VL	463.3	1.57	VL	468.5	1.73	VL
468.9	1.73	VL	468.5	1.70	VL	473.3	1.86	VL
472.9	1.85	VL	473	1.82	VL	448.7	1.29	LCST [b]
437.1	1.08	LCST [b]	442.2	1.16	LCST [b]	453.5	1.99	LL
443.5	2.06	LL	443.5	1.38	LL	458.7	2.75	LL
448.6	2.81	LL	448.3	2.07	LL	463.4	3.41	LL
453.4	3.48	LL	453.7	2.86	LL	468.5	4.08	LL
458.8	4.23	LL	458.2	3.47	LL	473.3	4.74	LL
463.7	4.89	LL	463.3	4.17	LL			
468.7	5.55	LL	468.5	4.86	LL			
473.1	6.12	LL	473.1	5.15	LL			

[a] VL refers to the vapor-liquid phase transition, and LL refers to the liquid-liquid phase transition; [b] The LCST point is the intersection of polynomial fits to LL and VL phase behavior data; [c] At these polymer concentrations, only the L (liquid) → LL (liquid-liquid) phase transition data were measured. [d] The combined standard uncertainties are $u_c(w_P) = 0.0008$ g·g^{-1}, $u_c(T) = 0.35$ K, $u_c(P) = 0.051$ MPa for the L → VL and LL → VLL transitions, and $u_c(P) = 0.092$ MPa for the L → LL transition.

S.2 Uncertainty analysis

S2.1 Uncertainty analysis method

(1) Calculation of the combined standard uncertainty

All uncertainty calculations performed in this study followed the Guide to the Expression of Uncertainty in Measurement (GUM) [36]. According to the uncertainty propagation law described in Section 5.2.2 of the GUM, the uncertainties of the input quantities X_i were propagated to the output quantity, and the corresponding mathematical relationship is given in Equation (S-1):

$$u_c(y)=\sqrt{\sum_{i=1}^{N}\left(\frac{\partial f}{\partial x_i}\right)^2 u^2(x_i)+2\sum_{i=1}^{N-1}\sum_{j=i+1}^{N}\left(\frac{\partial f}{\partial x_i}\right)\left(\frac{\partial f}{\partial x_j}\right)u(x_i,x_j)} \quad \text{(S-1)}$$

In this equation, y denotes the estimated value of the output quantity Y, x_i denotes the estimated value of the input quantity X_i, and f represents the functional relationship between the input quantities and the output quantity. $u_c(y)$ denotes the combined standard uncertainty of y, $u(x_i)$ denotes the standard or combined uncertainty of x_i, and $u(x_i, x_j)$ denotes the covariance between the input quantities x_i and x_j.

(2) Standard uncertainty of the estimated input quantities

The standard uncertainty of the estimated input quantities, $u(x_i)$, can be obtained using two types of approaches. The first is based on the statistical analysis of a series of observations of x_i (Type A evaluation), in which case the uncertainty is usually expressed as the experimental standard deviation of the mean. The second is based on evaluation using experience or other available information (Type B evaluation). In many cases, it is difficult to obtain a sufficiently large number of measurements required for a Type A evaluation; therefore, a Type B approach can be adopted to convert empirical or non-statistical information into standard uncertainty. In this study, three different Type B evaluation methods were employed and are summarized in Table S3. In all cases, the input quantity X was assumed to follow a rectangular distribution, i.e., the probability of values within the specified interval in the table was considered to be equal.

Table S3. Methods for determining standard uncertainty from non-statistical definitions

Informal definition of the uncertainty of x	Interval of X	Standard uncertainty $u(x)$
The error of x does not exceed $\pm x_E$	$X \in [x\text{-}x_E; x\text{+}x_E]$	$u(x) = \frac{x_E}{\sqrt{3}}$
x is greater than or equal to $x–x_L$	$X \in [x–x_L; x]$	$u(x) = \frac{x_L}{\sqrt{12}}$
x is less than or equal to $x + x_U$	$X \in [x; x\text{+}x_U]$	$u(x) = \frac{x_U}{\sqrt{12}}$
The range of x does not exceed x_R	$X \in \left[x-\frac{x_R}{2}; x+\frac{x_R}{2}\right]$	$u(x) = \frac{x_R}{\sqrt{12}}$

(3) Covariance between the estimated input quantities

The evaluation of the covariance $u(x_i, x_j)$ between correlated input quantities is relatively complex. In this study, all input quantities were treated as statistically independent, and covariance terms were neglected in the uncertainty propagation. A conservative assessment showed that inclusion of covariance would not significantly affect the combined uncertainty.

(4) Effective degrees of freedom

The effective degrees of freedom for the Type A evaluation, $v_{\mathrm{eff,A}}$, can be calculated using Equation (S-2), as given in Section G.4.1 of the GUM:

$$v_{\mathrm{eff,A}} = \left[\sum_i^n \left(\frac{\partial y}{\partial x_i}\right)^2 u^2(x_i)\right]^2 \left[\sum_i^n \left(\frac{\partial y}{\partial x_i}\right)^4 \left(\frac{u^4(x_i)}{v_i}\right)\right]^{-1} \quad \text{(S-2)}$$

According to Section G.4.3 of the GUM, the effective degrees of freedom of Type B standard uncertainties are considered to be infinite.

S2.2 Uncertainty of temperature

The combined standard uncertainty of temperature, $u_c(T)$, was composed of the following contributions: (1) the uncertainty $u(T_{\mathrm{ind}})$ arising from the accuracy p, resolution r, and hysteresis h of the temperature indicator; (2) the calibration uncertainty of the temperature sensor, $u(\varepsilon_T)$; (3) the uncertainty associated with temperature fluctuations during measurement, $u(\delta_{\mathrm{fluct}})$; and (4) the uncertainty related to the temperature measurement method, $u(\delta_{\mathrm{method}})$. The combined uncertainty was evaluated according to the relationship given in Equation (S-3):

$$u_c(T) = \sqrt{u^2(T_{\mathrm{ind}}) + u^2(\varepsilon_T) + u^2(\delta_{\mathrm{fluct}}) + u^2(\delta_{\mathrm{method}})} \quad \text{(S-3)}$$

The uncertainty arising from the accuracy (p = 0.5 °C), resolution (r = 0.1 °C), and hysteresis (h = 0.002 °C) of the temperature indicator was evaluated as $u^2(T_{\text{ind}}) = p^2/3 + r^2/12 + h^2/12$ = 0.29 °C; The calibration uncertainty was $u(\varepsilon_T)$ = 0.19 °C; During phase-transition temperature measurements, the temperature fluctuation was less than 0.2 °C, and thus $u(\delta_{\text{fluct}}) = 0.2/\sqrt{12}$ = 0.06 °C; Since the temperature probe was inserted directly into the fluid, the uncertainty associated with the measurement method was considered negligible, i.e., $u(\delta_{\text{method}})$ = 0. Therefore, the combined standard uncertainty of temperature was $u_c(T)$ = 0.35 °C.

S2.3 Uncertainty of component

To determine the uncertainty of the components, Equation (S-1) was applied to Equations (S-4)–(S-9) according to the properties of the different systems, yielding the corresponding equations used to calculate the component uncertainties.

(1) For the (n-pentane/n-hexane/iso-hexane + polymer) systems,

$$w_{\text{P}} = \frac{m_{\text{P}}}{m_{\text{P}} + m_{\text{sol}}} \tag{S-4}$$

$$u_{\text{c}}\left(w_{\text{P}}\right) = \sqrt{\left(\frac{\partial w_{\text{P}}}{\partial m_{\text{P}}}\right) u^2\left(m_{\text{P}}\right) + \left(\frac{\partial w_{\text{P}}}{\partial m_{\text{sol}}}\right) u^2\left(m_{\text{sol}}\right)} \tag{S-5}$$

where the solvent mass m_{sol} is the mass of the pure alkane solvent, and $u(m_{\text{P}})$ and $u(m_{\text{sol}})$ were calculated according to Tables S11 and S12, respectively.

(2) For the (α-olefin + n-hexane + polymer) systems,

$$w_{\text{P}} = \frac{m_{\text{P}}}{m_{\text{P}} + m_{\text{n-hexane}} + m_{\alpha\text{-olefin}}} \tag{S-6}$$

$$w_{\alpha\text{-olefin}} = \frac{m_{\alpha\text{-olefin}}}{m_{\alpha\text{-olefin}} + m_{\text{n-hexane}}} \tag{S-7}$$

$$u_{\text{c}}\left(w_{\text{P}}\right) = \sqrt{\left(\frac{\partial w_{\text{P}}}{\partial m_{\text{P}}}\right) u^2\left(m_{\text{P}}\right) + \left(\frac{\partial w_{\text{P}}}{\partial m_{\text{n-hexane}}}\right) u^2\left(m_{\text{n-hexane}}\right) + \left(\frac{\partial w_{\text{P}}}{\partial m_{\alpha\text{-olefin}}}\right) u^2\left(m_{\alpha\text{-olefin}}\right)} \tag{S-8}$$

$$u_{\text{c}}\left(w_{\alpha\text{-olefin}}\right) = \sqrt{\left(\frac{\partial w_{\alpha\text{-olefin}}}{\partial m_{\alpha\text{-olefin}}}\right) u^2\left(m_{\alpha\text{-olefin}}\right) + \left(\frac{\partial w_{\alpha\text{-olefin}}}{\partial m_{\text{n-hexane}}}\right) u^2\left(m_{\text{n-hexane}}\right)} \tag{S-9}$$

where the solvent mass $m_{\text{n-hexane}}$ is the mass of pure n-hexane, and $u(m_{\text{P}})$, $u(m_{\text{n-hexane}})$, and $u(m_{\alpha\text{-olefin}})$ were calculated according to Tables S11 and S12, respectively.

S2.4 Uncertainty of pressure

The combined standard uncertainty of pressure measurement, $u_c(P_{meas})$ was composed of the following contributions: (1) the uncertainty $u(P_{ind})$ arising from the accuracy (p = 0.028 MPa) and resolution (r = 0.01 MPa) of the pressure indicator; (2) the pressure calibration uncertainty $u(\varepsilon_P)$; (3) the uncertainty due to pressure fluctuations during measurement $u(\delta_{fluct})$; (4) the uncertainty associated with hysteresis, $u(\delta_{hyst})$; and (5) the uncertainty related to the pressure measurement method, $u(\delta_{method})$. The combined uncertainty was evaluated according to the relationship given in Equation (S-10)：

$$u_c(P_{meas}) = \sqrt{u^2(P_{ind}) + u^2(\varepsilon_P) + u^2(\delta_{fluct}) + u^2(\delta_{hyst}) + u^2(\delta_{method})} \quad \text{(S-10)}$$

The uncertainty of the pressure sensor, $u(P_{ind})$, was determined based on the uncertainties associated with the sensor accuracy and resolution as specified in the manufacturer's datasheet.

$$u(P_{ind}) = \sqrt{\left(\frac{0.03}{\sqrt{3}}\right)^2 + \left(\frac{0.01}{\sqrt{12}}\right)^2} = 0.018\ \text{MPa} \quad \text{(S-11)}$$

The uncertainty associated with pressure measurement fluctuations was estimated using the following equation, in which the pressure fluctuation measured in the phase-equilibrium cell used in the experiments was approximately 0.1 MPa.

$$u(\delta_{fluct}) = \sqrt{\left(\frac{0.1}{\sqrt{12}}\right)^2} = 0.029\ \text{MPa} \quad \text{(S-12)}$$

The uncertainty associated with hysteresis, $u(\delta_{hyst})$, was determined from the maximum hysteresis observed in the pressure calibration data, which was 0.03 MPa.

$$u(\delta_{hyst}) = \sqrt{\left(\frac{0.03}{\sqrt{3}}\right)^2} = 0.009\ \text{MPa} \quad \text{(S-13)}$$

Since the diaphragm of the pressure sensor was in direct contact with the fluid, the uncertainty introduced by the pressure measurement method was considered negligible. According to the calibration data, the pressure calibration uncertainty was evaluated based on an accuracy class of 0.1% of full scale (FS). Taking a full-scale pressure of 20 MPa as the reference, the calibration uncertainty $u(\varepsilon_P)$ was 0.02 MPa. Therefore, the uncertainty of the pressure measurement was:

$$u_c\left(P_{meas}\right)=\sqrt{\left(\frac{0.03}{\sqrt{3}}\right)^2+\left(\frac{0.01}{\sqrt{3}}\right)^2+\left(\frac{0.1}{\sqrt{12}}\right)^2+\left(\frac{0.03}{\sqrt{3}}\right)^2+\left(\frac{0.02}{\sqrt{3}}\right)^2}=0.04\text{ MPa} \quad \text{(S-14)}$$

The standard uncertainty of the phase-transition pressure, $u_c(P)$, was subsequently calculated using Equation (S-15):

$$u_c^2\left(P\right)=u_c^2\left(P_{meas}\right)+\left(\frac{\partial P}{\partial T}\right)^2 u_c^2\left(T\right)+\left(\frac{\partial P}{\partial w_P}\right)^2 u_c^2\left(w_P\right)+\left(\frac{\partial P}{\partial w_{\alpha\text{-olefin}}}\right)^2 u_c^2\left(w_{\alpha\text{-olefin}}\right) \quad \text{(S-15)}$$

Here, $u_c(P_{meas})$, $u_c(T)$, $u_c(w_P)$, $u_c(w_{\alpha\text{-olefin}})$ and were calculated as described in Tables S10, S9 and S12 in Section S2.7, respectively. The values of $(\partial P/\partial T)$, $(\partial P/\partial w_P)$, and $(\partial P/\partial w_{\alpha\text{-olefin}})$ depend on the nature of the system and the type of phase transition, as listed in Table S4.

Table S4. Dependence of the transition pressures P on the temperature T, polymer mass fraction w_P, and α-olefin mass fraction $w_{\alpha\text{-olefin}}$.

System	$(\partial P/\partial T)$/MPa·°C^{-1}		$(\partial P/\partial w_P)$/MPa·g·g^{-1}		$(\partial P/\partial w_{\alpha\text{-olefin}})$/MPa·g·g^{-1}	
	VL, VLL[a]	LL[b]	VL, VLL[a]	LL[b]	VL, VLL[a]	LL[b]
n-hexane+POE96k-10	0.018[c]	0.138[d]	0[e]	0.943[f]	—	—
ethylene+n-hexane+POE96k-10	0.018[c]	0.138[d]	0[e]	0.943[f]	32.99	142[h]
1-butene+n-hexane+POE96k-10	0.018[c]	0.138[d]	0[e]	0.943[f]	0[g]	25[h]
1-hexene+n-hexane+POE96k-10	0.018[c]	0.138[d]	0[e]	0.943[f]	0[g]	0.4[h]
1-octene+n-hexane+POE96k-10	0.018[c]	0.138[d]	0[e]	0.943[f]	0[g]	-11[h]

[a] VL or VLL refers to the vapor-liquid phase transition.
[b] LL refers to the liquid-liquid phase transition.
[c] Estimated from the average of the slopes of all the VL and VLL lines in Figure 3 and Figure 5(a)-(d).
[d] Estimated from the average of the slopes of all the LL lines in Figure 3 and Figure 5(a)-(d).
[e] The VL and VLL lines are essentially unaffected by the polymer.
[f] Estimated from the slopes of the isotherms on the Pw-plot in Figure 4.
[g] The larger comonomers have negligible effects on the VL and VLL lines. [h] Taken from Figure 7.

S2.5 Summary of uncertainties according to system and phase transition

Table S5. Summary of standard uncertainties u_c of the reported temperatures, pressures, polymer mass fractions w_P, and α-olefin mass fractions $w_{\alpha\text{-olefin}}$. [a]

System	$u_c(T)$/K	$u_c(P)$/MPa		$u_c(w_P)$/g·g^{-1}	$u_c(w_{\alpha\text{-olefin}})$/g·g^{-1}
		VL/VLL[b]	LL[c]		
n-hexane+POE96k-10	0.35	0.051	0.092	0.0008	—
ethylene+n-hexane+POE96k-10	0.35	0.058	0.135	0.0005	0.0008
1-butene+n-hexane+POE96k-10	0.35	0.051	0.113	0.0004	0.0009
1-hexene+n-hexane+POE96k-10	0.35	0.051	0.092	0.0004	0.0016
1-octene+n-hexane+POE96k-10	0.35	0.051	0.092	0.0004	0.0022

[a] For a conservative estimate, the uncertainty was taken as the maximum value calculated among all systems.
[b] VL or VLL refers to the vapor-liquid phase transition; [c] LL refers to the liquid-liquid phase transition.

S2.6 Uncertainty analysis of LCST determination

(1) Source of Uncertainty

The uncertainty associated with the determination of the lower critical solution temperature (LCST) mainly originates from the statistical uncertainty of the fitted phase-boundary parameters, rather than from instrumental temperature or pressure measurement errors. Since the LCST is defined as the intersection point of two empirically fitted phase boundaries in the pressure–temperature (P–T) space, its location is governed by the uncertainties of the fitted polynomial coefficients. Instrumental uncertainties in temperature and pressure measurements are implicitly reflected in the regression residuals and, therefore, are not treated as independent dominant sources of uncertainty in the present analysis.

(2) Monte Carlo–Based Uncertainty Propagation for LCST Temperature

To quantify the uncertainty of the LCST temperature, a Monte Carlo–based error propagation approach was employed. For each phase boundary, the quadratic polynomial coefficients *a*, *b*, and *c*, together with their corresponding standard errors, were obtained from least-squares regression of the experimental phase-transition data. Each coefficient was assumed to follow a normal distribution centered at its fitted value, with a variance equal to the square of its standard error.

Random sampling of the polynomial coefficients was performed independently for the two phase boundaries. For each Monte Carlo iteration, a pair of quadratic equations describing the phase boundaries was constructed using the sampled coefficients. The LCST temperature was calculated by solving the intersection condition:

$$P_1\left(T_{\mathrm{LCST}}\right) = P_2\left(T_{\mathrm{LCST}}\right) \tag{S-16}$$

which leads to a quadratic equation in temperature. Among the two mathematical solutions of the resulting quadratic equation, only the physically meaningful root located within the experimentally investigated temperature range was retained.

This procedure was repeated for a total of 10,000 Monte Carlo iterations, yielding a statistical distribution of LCST temperatures. The mean value of this distribution was taken as the reported LCST temperature, while the standard deviation was used to quantify the standard uncertainty of the LCST temperature.

The mean value of the resulting LCST distribution was taken as the reported LCST temperature, while the standard deviation of the distribution was used to quantify the standard uncertainty of the LCST. The expanded uncertainty was calculated by applying a coverage factor of $k = 2$, corresponding to a confidence level of approximately 95%, in accordance with the Guide to the Expression of Uncertainty in Measurement (GUM).

(3) Uncertainty of LCST Pressure

For each Monte Carlo iteration, the LCST pressure was calculated by substituting the corresponding T_{LCST} into one of the sampled phase-boundary polynomials. Since the two phase boundaries intersect at the LCST by definition, either polynomial yields an identical pressure within numerical precision. In this work, the quadratic polynomial fitted to the L → LL phase boundary was used to calculate the LCST pressure.

By repeating this calculation for all Monte Carlo iterations, a statistical distribution of LCST pressures was obtained. The mean value of the distribution was taken as the reported LCST pressure, and the standard deviation was used to quantify its standard uncertainty. The expanded uncertainty of the LCST pressure was calculated using a coverage factor of $k = 2$, corresponding to a confidence level of approximately 95%, in accordance with the Guide to the Expression of Uncertainty in Measurement (GUM).

(4) Illustrative Calculation Example

To facilitate reproducibility, an illustrative example is provided to demonstrate the computational workflow for obtaining T_{LCST} and P_{LCST} together with their uncertainties using the Monte Carlo procedure described above. As an illustrative case, the (n-hexane + POE96k-10) system was selected, and the two phase boundaries were represented by quadratic polynomials:

$$P_1(T) = a_1 T^2 + b_1 T + c_1 \ (\mathrm{L} \rightarrow \mathrm{LL}) \tag{S-17}$$

$$P_2(T) = a_2 T^2 + b_2 T + c_2 \ (\mathrm{L} \rightarrow \mathrm{VL} \ / \ \mathrm{LL} \rightarrow \mathrm{VLL}) \tag{S-18}$$

where the fitted coefficients and their standard errors (1σ) are summarized in Table S6.

Table S6. Illustrative polynomial coefficients (mean ± standard error) used in the Monte Carlo example.

Boundary	a/(MPa·K^{-2})	b/(MPa·K^{-1})	c/MPa
$P_1(T)$ (L → LL)	(−4.927 ± 0.0001)×10^{-4}	0.588 ± 0.0480	−162.5 ± 10.9
$P_2(T)$ (L → VL / LL → VLL)	(1.023 ± 0.00001)×10^{-4}	−0.071 ± 0.007	12.28 ± 1.62

In each Monte Carlo iteration, a set of coefficients $\{a_1, b_1, c_1, a_2, b_2, c_2\}$was randomly sampled from normal distributions defined by the fitted means and standard errors. The LCST temperature was obtained by solving the intersection condition $P_1(T_{\mathrm{LCST}}) = P_2(T_{\mathrm{LCST}})$, i.e.,

$$(a_1 - a_2)T_{\mathrm{LCST}}^2 + (b_1 - b_2)T_{\mathrm{LCST}} + (c_1 - c_2) = 0 \tag{S-19}$$

Among the two mathematical solutions, only the root within the experimentally investigated temperature window was retained. The corresponding LCST pressure was then calculated by substituting T_{LCST} into the sampled L → LL polynomial, i.e., $P_{\mathrm{LCST}} = P_1(T_{\mathrm{LCST}})$.

After 10,000 iterations, the Monte Carlo distributions yielded combined standard uncertainties of $u_c(T_{\mathrm{LCST}}) = 2.75$ K and $u_c(P_{\mathrm{LCST}}) = 0.34$ MPa. Accordingly, the expanded uncertainties at a 95% confidence level were $U_{95}(T_{\mathrm{LCST}}) = 5.50$ K and $U_{95}(P_{\mathrm{LCST}}) = 0.68$ MPa, corresponding to the confidence intervals $T_{\mathrm{LCST}} \pm 5.50$ K and $P_{\mathrm{LCST}} \pm 0.68$ MPa, respectively.

Table S7. Uncertainty and 95% confidence intervals of LCST temperature and pressure for different (α-olefin + n-hexane + POE96k-10) systems.

System	$u_c(T_{\mathrm{LCST}})$/K	$u_c(P_{\mathrm{LCST}})$/MPa	$U_{95}(T_{\mathrm{LCST}})$/K	$U_{95}(P_{\mathrm{LCST}})$/MPa
n-hexane+POE96k-10	2.75	0.34	5.50	0.68
ethylene+n-hexane+POE96k-10	4.40	0.39	8.80	0.78
1-butene+n-hexane+POE96k-10	4.34	0.48	8.68	0.96
1-hexene+n-hexane+POE96k-10	4.55	0.47	9.10	0.94
1-octene+n-hexane+POE96k-10	4.52	0.40	9.04	0.80

Table S8. Quadratic phase-boundary fitting parameters (mean ± standard error) for the determination of LCST in the (n-hexane + POE96k-10) and (α-olefin + n-hexane + POE96k-10) systems.

System	Boundary	a/(MPa·K^{-2})	b/(MPa·K^{-1})	c/MPa
n-hexane +POE96k-10	L → LL	$(-4.927 \pm 0.0001)\times10^{-4}$	0.588 ± 0.0480	−162.50 ± 10.90
	L → VL/LL → VLL	$(1.023 \pm 0.00001)\times10^{-4}$	−0.071 ± 0.007	12.28 ± 1.62
ethylene + n-hexane + POE96k-10	L → LL	$(-3.936 \pm 0.0001)\times10^{-4}$	0.492 ± 0.050	−136.98 ± 11.25
	L → VL/LL → VLL	$(1.067 \pm 0.0001)\times10^{-4}$	−0.074 ± 0.017	13.29 ± 3.64
1-butene + n-hexane + POE96k-10	L → LL	$(-3.961 \pm 0.0001)\times10^{-4}$	0.486 ± 0.040	−129.49 ± 8.88
	L → VL/LL → VLL	$(1.271 \pm 0.0001)\times10^{-4}$	−0.084 ± 0.023	14.31 ± 5.06
1-hexene + n-hexane + POE96k-10	L → LL	$(-3.531 \pm 0.0001)\times10^{-4}$	0.461 ± 0.125	−132.03 ± 28.55
	L → VL/LL → VLL	$(1.277 \pm 0.0001)\times10^{-4}$	−0.091 ± 0.021	16.45± 4.46
1-octene + n-hexane + POE96k-10	L → LL	$(-2.458 \pm 0.0003)\times10^{-4}$	0.349 ± 0.100	−81.05 ± 3.29
	L → VL/LL → VLL	$(1.121 \pm 0.0001)\times10^{-4}$	−0.081 ± 0.018	14.76 ± 3.91

S2.7 Detailed Uncertainty Report

Table S9. Detailed uncertainty analysis for phase transition temperature [a]

Measurement model	$T = T_{ind} + \varepsilon_T + \delta_{fluct} + \delta_{method}$			$u_c(T)$	0.35 °C	
Uncertainty component	Source and evaluation of standard uncertainty $u(x_i)$	Type	Degrees of freedom v_i	$(\partial T/\mathrm{d}x_i)$	$u(x_i)$	Unit
$u(T_{ind})$	Uncertainty arising from the accuracy (p = 0.5 °C), resolution (r = 0.1 °C), and hysteresis (h = 0.002 °C) of the temperature indicator, evaluated as $u^2(T_{ind}) = p^2/3 + r^2/12 + h^2/12$.	Type B	∞	1	0.29	°C
$u(\varepsilon_T)$	Calibration uncertainty of the temperature indicator, originating from accuracy, non-repeatability, and offset. It was determined by calibration based on 20 repeated measurements at four temperature points between 0 and 200 °C.	Type A	50	1	0.19	°C
$u(\delta_{fluct})$	Uncertainty due to temperature fluctuations during phase-transition measurements. For at least three repeated measurements, the temperature fluctuation was less than 0.2 °C; thus, $u(\delta_{fluct}) = 0.2/\sqrt{12}$.	Type B	∞	1	0.06	°C
$u(\delta_{method})$	Method-related uncertainty due to the possible deviation between the probe temperature and the actual fluid temperature. Since the probe was fully immersed in the fluid, this uncertainty was considered negligible.	Type B	∞	1	0	°C

[a] All input quantities were treated as statistically independent, and covariance terms were neglected in the uncertainty propagation. A conservative assessment showed that inclusion of covariance would not significantly affect the combined uncertainty.

Table S10. Detailed uncertainty analysis for pressure measurement [a]

Measurement model	$P_{meas} = P_{ind} + \varepsilon_P + \delta_{fluct} + \delta_{hyst} + \delta_{method}$			$u_c(P_{meas})$	0.03 MPa[b] / 0.04 MPa[c]	
Uncertainty component	Source and evaluation of standard uncertainty $u(x_i)$	Type	Degrees of freedom v_i	$(\partial P_{meas}/\mathrm{d}x_i)$	$u(x_i)$	Unit
$u(P_{ind})$	The uncertainty arising from the accuracy (p = 0.03 MPa) and resolution (r = 0.01 MPa) of the pressure indicator was evaluated as $u^2(P_{ind}) = p^2/3 + r^2/12$.	Type B	∞	1	0.018	MPa
$u(\varepsilon_P)$	The calibration uncertainty of the pressure sensor indicator originated from the sensor characteristics, offset, accuracy, short-term drift, and temperature effects. The correction values were determined from 280 calibration experiments conducted using an independently calibrated deadweight pressure balance ($u_c(P_{ref}$ = 0.006 MPa))at five temperatures between 50 and 170 °C and at fourteen pressures between 0.5 and 20 MPa (absolute).	Type A	50	1	0.02	MPa
$u(\delta_{fluct})$	The uncertainty due to pressure fluctuations during measurement was evaluated as follows. For the L → VL and LL → VLL phase transitions, at least three repeated measurements were performed, and the pressure fluctuation was less than 0.03 MPa; thus, $u(\delta_{fluct}) = 0.03/\sqrt{12}$; For the L → LL phase transition, the pressure fluctuation was less than 0.1 MPa, and $u(\delta_{fluct}) = 0.1/\sqrt{12}$.	Type B	∞	1	0.009[b]/0.029[c]	MPa
$u(\delta_{hyst})$	The standard deviation of the hysteresis band observed during the calibration experiments was evaluated using the method proposed in the GUM. Since the maximum hysteresis was 0.03 MPa, the corresponding uncertainty was estimated as $u(\delta_{hyst}) = 0.03/\sqrt{12}$ = 0.009 MPa.	Type B	∞	1	0.009	MPa
$u(\delta_{method})$	Since the diaphragm of the pressure sensor was in direct contact with the fluid, the uncertainty introduced by the pressure measurement method was considered negligible.	Type B	∞	1	0	MPa

[a] All input quantities were treated as statistically independent, and covariance terms were neglected in the uncertainty propagation. A conservative assessment showed that inclusion of covariance would not significantly affect the combined uncertainty. [b] L → VL and LL → VLL phase transitions. [c] L → LL phase transition.

Table S11. Detailed uncertainty analysis of the polymer loading mass

Measurement model	$m_P = m_i - m_f$			$u_c(m_P)$	0.00056 g	
Uncertainty component [b]	Source and evaluation of standard uncertainty $u(x_i)$	Type	Degrees of freedom v_i	$(\partial m_P/dx_i)$	$u(x_i)$	Unit
$u(m_i)$ [a]	Due to the uncertainty in the accuracy of the analytical balance, u_c(ME XPR226DRQ/AC) = 0.0002 g, the uncertainty of the solid mass m_i before the polymer was added to the phase-equilibrium cell.	Type A	4	1	0.0002	g
$u(m_f)$ [a]	After the polymer was added to the phase-equilibrium cell, the uncertainty of the measured mass m_f was evaluated. This uncertainty includes contributions from the accuracy of the analytical balance and the sample purity, i.e., $u^2(m_f) = u^2$(ME XPR226DRQ/AC) + $u^2(\delta_{\text{purity}})$ (see Section F.2.4.5 of the GUM). The estimation of $u(\delta_{\text{purity}})$ is described as follows. Assuming that the mass fraction of impurities in the polymer is p, the mass of impurities transferred into the phase-equilibrium cell is $\delta_{\text{purity}} = p \times m_P$. This quantity essentially represents an uncorrected systematic effect (see Section F.2.4.5 of the GUM); therefore, $u^2\left(\delta_{\text{purity}}\right) = \frac{1}{m_P}\int_0^{m_P}\left[pM - \frac{1}{m_P}\int_0^{m_P} pMdM\right]^2 dM = \frac{p^2 m_P^2}{12}$ For POE96k-10, $p = 0$.	Type B	∞	−1	0.0002	g

[a] The same electronic analytical balance (METTLER XPR226DRQ/AC) was used. Calibration was performed using five reference masses over the full weighing range of the analytical balance, and u_c(ME XPR226DRQ/AC) = 0.0002 g. [b] Since the electronic analytical balance was zeroed between two measurements, the covariance between m_i and m_f was zero; therefore, m_i and m_f are independent.

Table S12. Detailed Uncertainty Report for the Composition of Solvent Mixture Loaded

Measurement model	$w_{\alpha\text{-olefin}} = (m_1\text{-}m_0)/(m_2\text{-}m_0)$			$u_c(w_{\alpha\text{-olefin}})$	0.0002 $g \cdot g^{-1}$	
Uncertainty component [b]	Source and evaluation of standard uncertainty $u(x_i)$	Type	Degrees of freedom v_i	$(\partial w_\alpha/dx_i)$ [a]	$u(x_i)$	Unit
$u(m_0)$	Due to the uncertainty of the analytical balance u_c(ME XPR226DRQ/AC) = 0.0002 g, there is an associated uncertainty in the mass of the empty vial m_0 prior to charging the α-olefin.	Type A	4	$(m_1\text{-}m_2)/(m_2\text{-}m_0)^2$	0.0002	g
$u(m_1)$	The uncertainty in the mass of the vial m_1, which had been charged with a known amount of comonomer but had not yet been filled with *n*-hexane solvent, includes the following contributions: (i) the accuracy of the analytical balance; (ii) the possible evaporation loss of the comonomer during sample preparation, $u(\delta_{evap})$; and (iii) the purity of the comonomer, $u(\delta_{purity})$. Accordingly, the combined uncertainty can be expressed as $u^2(m_1) = u^2$(ME XPR226DRQ/AC) + $u^2(\delta_{evap}) + u^2(\delta_{purity})$。<br>The uncertainty $u(\delta_{evap})$ was estimated using a method reported in the literature. At 293 K, the evaporation rate of n-hexane is approximately $E = 5.69\times10^{-4}$ $kg \cdot m^{-2} \cdot s^{-1}$. Based on the geometry of the sample vial, the exposed surface area of *n*-hexane was estimated to be $A = 3.14\times10^{-4}$ m^2. The vial was exposed to the atmosphere for approximately $t = 300$ s. Therefore, the maximum possible mass loss during sample preparation can be estimated as $\delta_{evap} = t \times A \times E = 0.004$ g, and the corresponding standard uncertainty is $u(\delta_{evap}) = \delta_{evap}/\sqrt{12} = 0.001$ g. The evaporation rate of *n*-hexane was applied to all compounds used in this study, since *n*-hexane is the most volatile among them, thereby ensuring a conservative estimation of the uncertainty.<br>The estimation of $u(\delta_{purity})$ follows the same approach as that described in Table S.5. Accordingly, the uncertainty associated with comonomer purity is given by $u(\delta_{purity}) = p_1(m_1 - m_0)/\sqrt{12}$, where p_1 is the mass fraction of impurities in the comonomer.	Type B	∞	$1/(m_2\text{-}m_0)$	0.0072	g
$u(m_2)$	The uncertainty in the mass of the vial after charging with *n*-hexane m_2 was evaluated following the same procedure as for	Type B	∞	$-(m_1\text{-}m_2)/(m_2\text{-}m_0)^2$	0.1225	g

$u(m_1)$. However, in this case, $u(\delta_{\text{purity}}) = p_2(m_2 - m_1)/\sqrt{12}$, where p_2 is the mass fraction of impurities in *n*-hexane.

[a] In evaluating the partial derivatives, the following nominal and representative values were used: m_0 = 79.8254 g; m_1 = 88.5954 g; m_2 = 177.0032 g; p_1 = 0.0028 $g \cdot g^{-1}$ (1-octene); p_2 = 0.0048 $g \cdot g^{-1}$ (*n*-hexane); [b] Because the electronic analytical balance was tared (zeroed) between the three measurements, the covariances among m_0, m_1, and m_2 are zero; therefore, m_0, m_1 and m_2 are mutually independent.

Table S13. Detailed Uncertainty Report for the Mass of Solvent Mixture Loaded.

Measurement model	$m_{solv} = m_i - m_f$			$u_c(m_{solv})$	0.008 g	
Uncertainty component [b]	Source and evaluation of standard uncertainty $u(x_i)$	Type	Degrees of freedom v_i	$(\partial m_{solv}/dx_i)$ [a]	$u(x_i)$	Unit
$u(m_i)$	Due to the uncertainty of the analytical balance, u_c(ME XPR226DRQ/AC) = 0.0002 g, there is an associated uncertainty in the total mass m_i of the solvent (including the container) and the syringe prior to charging the solvent mixture into the phase-equilibrium cell.	Type A	4	1	0.0002	g
$u(m_f)$	The uncertainty in the total mass m_f of the remaining solvent (including the container) and the syringe after charging the solvent mixture into the phase-equilibrium cell includes the following contributions: (i) the accuracy of the analytical balance; and (ii) the possible solvent evaporation during solvent loading and degassing, $u(\delta_{evap})$. Accordingly, the combined uncertainty can be expressed as $u^2(m_f) = u^2$(ME XPR226DRQ/AC) + $u^2(\delta_{evap})$. The uncertainty $u(\delta_{evap})$ was estimated using a method reported in the literature. During solvent charging at 293 K, the evaporation rate of n-hexane is approximately E_1 = 5.69×10^{-4} kg·m^{-2}·s^{-1}; during vacuum degassing (assuming *n*-hexane is at its boiling point, 341 K), the evaporation rate is E_2 = 3.47×10^{-3} kg·m^{-2}·s^{-1}. Based on the geometry of the phase-equilibrium cell, the exposed surface area of *n*-hexane was estimated to be A = 7.85×10^{-5} m^2. The solvent charging time and degassing time were t_1 = 240 s and t_2 = 60 s, respectively. Therefore, the maximum possible solvent mass loss can be estimated as $\delta_{evap} = A(t_1E_1 + t_2E_2)$ = 0.0297 g, and the corresponding standard uncertainty is $u(\delta_{evap}) = \delta_{evap}/\sqrt{12}$ = 0.008 g.	Type B	∞	−1	0.008	g

[a] In evaluating the partial derivatives, the following nominal and representative values were used: m_i = 189.8573 g; m_f = 94.6795 g; [b] Because the electronic analytical balance was tared (zeroed) between the two measurements, the covariance between m_i and m_f is zero; therefore, m_i and m_f are independent.

Table S14. Detailed Uncertainty Report for the Mass of Gas (ethylene or 1-butene) Loaded.

Measurement model	$m_{gas} = m_i - m_f$			$u_c(m_{gas})$	0.0189 g	
Uncertainty component [a,b]	Source and evaluation of standard uncertainty $u(x_i)$	Type	Degrees of freedom v_i	$(\partial m_{gas}/dx_i)$ [a]	$u(x_i)$	Unit
$u(m_i)$	Due to the uncertainty of the analytical balance, u_c(ME XPR226DRQ/AC) = 0.0002 g, there is an associated uncertainty in the mass m_i of the gas cylinder prior to charging the gas into the phase-equilibrium cell.	Type A	4	1	0.0002	g
$u(m_f)$	The uncertainty in the mass of the gas cylinder after charging the gas into the phase-equilibrium cell m_f includes the following contributions: (i) the accuracy of the analytical balance; and (ii) the gas purity, $u(\delta_{purity})$. Accordingly, the combined uncertainty can be expressed as $u^2(m_f) = u^2$(ME XPR226DRQ/AC) $+ u^2(\delta_{purity})$. The estimation of $u(\delta_{purity})$ follows the same approach as that described in Table S.5. Thus, the uncertainty associated with gas purity is given by $u(\delta_{purity}) = p(m_i - m_f)/\sqrt{12}$, where p is the mass fraction of impurities in the gas.	Type B		−1	0.019	g

[a] In evaluating the partial derivatives, the following nominal and representative values were used: m_i = 199.6523 g; m_f = 193.1026 g; p = 0.01 g·g^{-1}. [b] Because the electronic analytical balance was tared (zeroed) between the two measurements, the covariance between m_i and m_f is zero; therefore, m_i and m_f are independent.

S.3 Robustness and sensitivity checks

S3.1 Influence of polynomial order

To examine the robustness of the LCST determination with respect to the polynomial order, the two phase boundaries were additionally fitted using a third-order polynomial in temperature, and the LCST was recalculated as the intersection of the two fitted curves. The differences between the LCST values obtained from the quadratic and cubic representations, ΔT_{LCST} and ΔP_{LCST}, were used as indicators of the sensitivity to model order.

$$\Delta T_{LCST} = \left| T_{LCST}^{(3rd)} - T_{LCST}^{(2nd)} \right| \tag{S-20}$$

$$\Delta P_{LCST} = \left| P_{LCST}^{(3rd)} - P_{LCST}^{(2nd)} \right| \tag{S-21}$$

As shown in Table S15, in all cases, the resulting changes in T_{LCST} and P_{LCST} were within the uncertainty bounds derived from the Monte Carlo analysis (Section S2.6), indicating that the LCST determination is not sensitive to the polynomial order within the investigated temperature range. Therefore, quadratic polynomials were adopted in this work as a parsimonious and robust representation of the experimental phase boundaries.

Table S15. Comparison of LCST temperature and pressure obtained from quadratic and cubic polynomial fits for different solvent systems.

System	T_{LCST}/K[a]	T_{LCST}/K[b]	Δ T_{LCST} /K	P_{LCST}/ MPa[a]	P_{LCST}/ MPa[b]	Δ P_{LCST} /MPa
n-hexane +POE96k-10 [c]	436.23	437.1	0.87	0.99	1.08	0.09
ethylene+n-hexane+POE96k-10 [d]	424.82	424.4	0.42	1.19	1.17	0.02
1-butene+n-hexane+POE96k-10 [e]	409.15	409.6	0.45	0.78	0.81	0.03
1-hexene+n-hexane+POE96k-10 [f]	433.24	431.5	1.74	0.95	0.92	0.03
1-octene+n-hexane+POE96k-10 [g]	448.14	447.4	0.74	1.03	0.99	0.04

[a] LCST temperature and pressure determined from quadratic polynomial fitting; [b] LCST temperature and pressure determined from cubic polynomial fitting.
[c] The polymer mass fraction was 0.1951 $g \cdot g^{-1}$; [d] The mass fractions of the polymer and ethylene were 0.0226 and 0.0105 $g \cdot g^{-1}$, respectively; [e] The mass fractions of the polymer and 1-butene were 0.0231 and 0.1140 $g \cdot g^{-1}$, respectively; [f] The mass fractions of the polymer and 1-hexene were 0.0234 and 0.4982 $g \cdot g^{-1}$, respectively; [g] The mass fractions of the polymer and 1-octene were 0.0228 and 0.2011 $g \cdot g^{-1}$, respectively;

S.4 Statistical analysis of concentration-dependent slopes

The dependence of the characteristic phase transition points on α-olefin mass fraction was quantified using linear regression analysis. For each characteristic point shown in Figure 7, the corresponding transition temperature or pressure was regressed as

a function of α-olefin mass fraction assuming a linear relationship. The regression parameters, including the slope and intercept, were determined using the ordinary least-squares method. To evaluate the reliability of the fitted slopes, confidence intervals were calculated based on the statistical uncertainties of the regression parameters. The results indicate that, within the investigated range of α-olefin mass fraction, the linear regression models provide an adequate description of the experimental data.

In the present system, the inclusion of confidence intervals is particularly important because the slopes describing the effects of α-olefin mass fraction are relatively small. By explicitly accounting for the regression uncertainty, this analysis enables a statistically meaningful assessment of whether the observed concentration-dependent trends exceed the experimental scatter. The resulting confidence intervals confirm that the linear trends reported in the main text are statistically resolvable within the experimental uncertainty. The detailed regression results, including the fitted parameters and their corresponding confidence intervals for each characteristic point, are summarized in Table S16. These statistical analyses support the linear relationships discussed in the main text.

Table S16. Linear regression slopes of characteristic points for different α-olefin + n-hexane + POE96k-10 systems. [a]

Characteristic point	$k \pm \Delta k$ (R^2) [b]			
	ethylene	1-butene	1-hexene	1-octene
$k(T_{LCST})$/K·g·g^{-1}	-841.9±308.6(0.986)	-173.5±35(0.996)	-2.211±1.320(0.904)	78.74±14.42(0.996)
$k(P_{LL}(475\ K))$/MPa·g·g^{-1}	141.81±45.75(0.989)	24.90±4.29(0.997)	0.451±0.283(0.919)	-11.24±2.23(0.996)
$k(T_{LL}(2\ MPa))$/K·g·g^{-1}	-993.5±252.0(0.993)	-176.20±30.53(0.997)	-2.406±1.458(0.928)	82.36±6.15(0.999)
$k(P_{LCST})$/MPa·g·g^{-1}	23.74±1.30(0.999)	-0.317±0.202(0.891)	0.0548±0.0332(0.894)	0.518±0.380(0.945)
$k(P_{VL}(430\ K))$/MPa·g·g^{-1}	38.9±4.5(0.999)	3.30±0.97(0.991)	0.092±0.072(0.847)	-0.768±0.386(0.973)
$k(T_{VL}(1.2\ MPa))$/K·g·g^{-1}	-2404±523(0.995)	-159±39(0.994)	-5.79±3.49(0.903)	46.5±12.4(0.992)

[a] Linear regressions were performed independently for each α-olefin + n-hexane + POE96k-10 system by regressing the characteristic transition temperature or pressure (dependent variable) against α-olefin mass fraction (independent variable). [b] The reported values correspond to the fitted slopes (k) with their associated confidence intervals (±Δk), and the values in parentheses denote the coefficient of determination (R^2).
